# Palliative and end-of-life care for people living with dementia in rural areas: A scoping review

**DOI:** 10.1371/journal.pone.0244976

**Published:** 2021-01-14

**Authors:** Valerie Elliot, Debra Morgan, Julie Kosteniuk, Melanie Bayly, Amanda Froehlich Chow, Allison Cammer, Megan E. O’Connell

**Affiliations:** 1 Canadian Centre for Health and Safety in Agriculture, University of Saskatchewan, Saskatoon, Saskatchewan, Canada; 2 School of Public Health, University of Saskatchewan, Saskatoon, Saskatchewan, Canada; 3 College of Pharmacy and Nutrition, University of Saskatchewan, Saskatoon, Saskatchewan, Canada; 4 Department of Psychology, University of Saskatchewan, Saskatoon, Saskatchewan, Canada; Nathan S Kline Institute, UNITED STATES

## Abstract

**Background and objectives:**

People living with dementia deserve to experience the benefits of receiving palliative care and end-of-life services and supports, yet they often do not receive this care compared to those with other terminal diseases. People living with dementia in rural areas often face additional challenges to accessing such care. The purpose of this scoping review was to systematically review and synthesize the literature on palliative and end-of-life care for people with dementia living in rural areas, and to identify and describe key findings and gaps in the literature.

**Methods:**

A collaborative research team approach was used in an iterative process across all stages of this review. Systematic, comprehensive searches were conducted across ten databases and eight targeted websites for relevant peer-reviewed, original research and other less formal literature, published in English, which yielded a total of 4476 results. After duplicate removal, screening, and review, 24 items were included for synthesis.

**Results:**

All items were described and illustrated by frequency distribution, findings were grouped thematically, and five key themes emerged, including: 1) Knowledge about dementia, 2) Availability, accessibility, and utilization of palliative and end-of-life care services and supports, 3) Decision-making about care, the value of a person-centered approach and collaborative support, 4) Perspectives on artificial nutrition, hydration, and comfort care, and 5) Quality of life and death. The main gap identified was literature pertaining to rural populations, especially from locations other than the United States. The influence of rurality on relevant findings was mixed across rural-urban comparison studies, as was the effect of sex and gender across the literature.

**Conclusions:**

Several areas were highlighted including the importance of increasing knowledge about dementia, having early conversations about advanced care and treatment options, providing a person-centered approach, and the potential for using technology to address rural access issues. These findings can be used to inform future research and policy and the development of services, supports, and strategies for rural people living with dementia. Further research is recommended.

## Introduction

Dementia is caused by a group of diseases that are typically progressive in nature, affect cognitive abilities (such as memory and decision-making) and behaviors, and affect the ability to perform activities of daily life [1]. Globally, it is estimated that over 50 million people are currently living with dementia [2]. This number is projected to increase by approximately 10 million each year, reaching 152 million by the year 2050 [2]. As a chronic, terminal disease, experiences, abilities, and needs vary across the different disease stages [2]. The World Health Organization describes palliative and end-of-life care as essential in advanced, late- or end-stage dementia [1].

Although there are several terms related to care provided during the end of life (for example hospice care, end-of-life care, and palliative care), these terms are at times used collectively to represent care that is intended to better the quality of life and death for people dealing with a terminal illness [3]. While palliative care and end-of-life care share common goals, most often palliative care can, and should, begin much earlier in the continuum of care than the end of life stage. In fact, advance planning for both palliative and end-of-life care can begin soon after diagnosis [4]. For the purposes of this review, the terms palliative care and end-of-life care are used the way they were referred to in the literature cited.

The Worldwide Hospice Palliative Care Alliance and the World Health Organization recognize that the benefits of palliative care extend to individuals living with chronic and life-limiting, terminal conditions [5]. However, there are many barriers to accessing palliative care for people with dementia such as misconceptions that dementia is not terminal, insufficient policy and resources, a lack of health professionals with training in palliative care and dementia, a deficit in advance directives regarding end-of-life care, and cultural and social beliefs related to death and dying [5–7]. It has been recognized that the palliative care needs of people with dementia are likely underestimated, under-assessed, and under-treated, on a global level [6]. In addition, people living in rural or remote areas more often consist of older populations [8] and experience additional unique barriers to accessing care and services [9] compared to their urban counterparts [8,9].

The purpose of this review was to scope the literature pertaining to palliative and end-of-life care for people with dementia and their families who live in rural areas. Specifically, to comprehensively review and summarize the evidence regarding palliative and end-of-life care for people with dementia living in rural areas, identify key findings and gaps in the literature, and make recommendations for future research. The findings from this review contribute to existing knowledge on palliative/end-of-life care for people with dementia and their families while incorporating unique challenges or facilitators associated with living rurally.

## Methods

The protocol used to conduct this scoping review was based on the methodological framework of Arksey and O'Malley [10], built on by Levac et al [11], and later revised and updated by the Joanna Briggs Institute [12,13]. The protocol was developed *a priori* and was not registered. The five-step framework [10] included the following: (i) identifying the research questions, (ii) identifying the relevant studies, (iii) study selection, (iv) data charting, and (v) collating, summarizing, and reporting the results. A collaborative research team approach was used in the iterative process of developing the research questions, search strategies, and data extraction form. Further, the Preferred Reporting Items for Systematic reviews and Meta-Analyses extension for Scoping Reviews (PRISMA-ScR) [14] checklist was used post-hoc as a rigorous reporting guideline (S1 Appendix).

### Step one: Identifying the research questions

The following research questions were explored: What were the main themes identified across the literature pertaining to dementia-related palliative and end of life care? Were there gaps in knowledge identified in the literature? In literature that reported on both rural and urban populations, were there any differences in findings between rural and urban? Were there any differences in findings based on individual characteristics such as sex and gender or study characteristics such as the country in which the literature originated?

### Step two: Identifying the relevant studies

A broad search strategy was designed with the guidance of a university health sciences librarian. Searches were conducted across four scholarly databases (MEDLINE, EMBASE, PSYCINFO, and CINAHL) that included key search terms related to “palliative” or “end-of-life”, “dementia”, and “rural”. Search strategies were customized to each specific database and are included in S2 Appendix. Final searches were completed on November 13, 2018, and restricted to English language only. The reference lists of studies selected for inclusion were hand-searched as a supplemental approach to identify any additional relevant studies.

In addition, grey literature and organization sites were searched. Grey search strategies are itemized and described in S3 Appendix.

### Step three: Study selection

All records were first imported to EndNote Desktop Version X8 (Clarivate Analytics, Philadelphia, United States) reference management software and then exported to DistillerSR (Evidence Partners, Ottawa, Canada) systematic review software. After deduplication, remaining items were screened for inclusion. Inclusion and exclusion criteria are itemized in Table 1.

**Table 1:** Inclusion and exclusion criteria.

Inclusion criteria	Exclusion criteria
• **Peer-reviewed literature:** original research • **Grey literature:** such as letters to the editor, opinion letters, commentaries, dissertations, study protocols, reviews, policy papers, reports, book chapters, and any non-peer-reviewed documents • English language only • Relevant to the research question(s) ○ Palliative and end-of-life care for people with AD or other dementias ○ Rural Settings (can include urban if rural-urban comparison is made)	• Publications or documents written in a language other than English • Publications or documents non-relevant to the research question(s) ○ Not pertaining to AD or other dementias ○ Not pertaining to palliative and end-of-life care ○ Non-rural settings (unless rural-urban comparison is made)

Forms were created and piloted in DistillerSR to screen the peer-reviewed literature across two levels. The first author screened all records and three coauthors each screened one-third of all records at the first level (title/abstract), followed by second level screening (full-text) conducted independently by two reviewers. The grey literature underwent an initial screen for relevance by the first author and all remaining records were screened in-depth by the first author and one coauthor. All types of original research methodologies and designs were eligible for inclusion. Inclusion criteria required the literature was relevant to the research questions regarding palliative or end-of-life care for rural people with dementia and their families (see Table 1). Unresolved screening conflicts were resolved by the second author.

The reference lists of peer-reviewed studies ultimately selected for inclusion were hand-searched and three additional studies were identified for inclusion.

### Step four: Data charting

The first author extracted key characteristics of the literature including author, year, country, objective/topic, study design/document type, and sample), findings, limitations, and conclusions relevant to this review. Additional information included whether the study was rural or rural-urban and the definition of rural used (when provided).

### Step five: Collating, summarizing, and reporting the results

Following the methodological framework of Arksey and O’Malley [10] and enhanced by Levac et al [11], this review incorporated both numerical summary data and thematic analysis. Peer-reviewed literature were collated by study methodology, publication year, and country and grey literature was collated by document type, publication year, and country. Thematic analysis of the main findings of the included literature was conducted. An inductive approach to thematic analysis was used to search for repeated patterns of meaning within the main findings of the charted data. The main findings were read and re-read thoroughly and analytically using a semantic approach to identify common, re-occurring explicit words or surface meanings in the data. Annotation and color highlighting were used to code the data as themes emerged and ultimately, data were named and grouped according to similarities or patterns.

## Results

**Fig 1:**
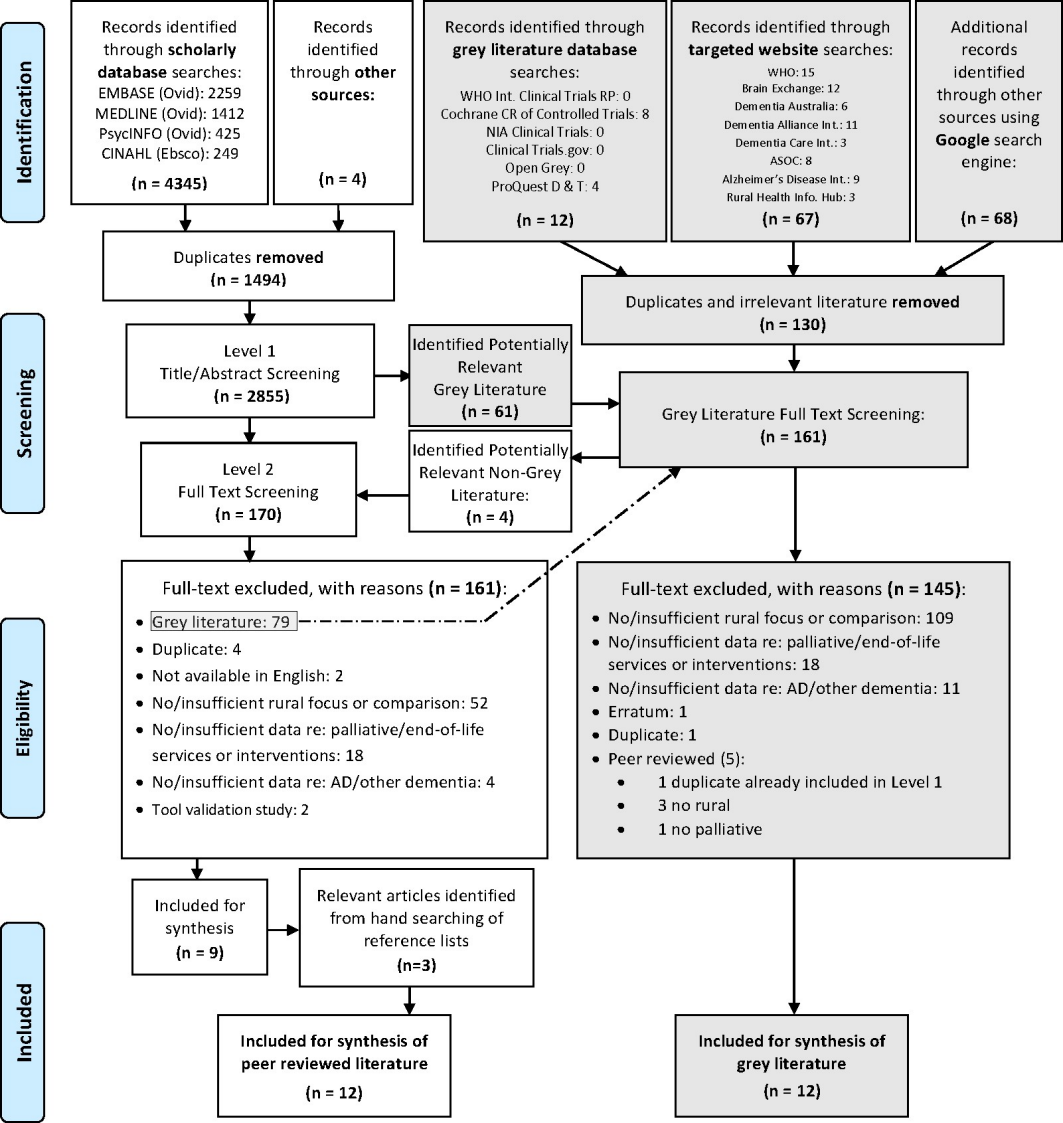
Modified PRISMA flow diagram.

Twenty-four items (*n* = 12 peer-reviewed research articles and *n* = 12 grey literature) were identified for inclusion in this review following the search and screen process represented in the modified PRISMA [15] flow diagram (Fig 1).

Overall, the literature included for synthesis used similar definitions or conceptualizations of the terms palliative care and end-of-life care as those presented earlier in this review.

**Fig 2:**
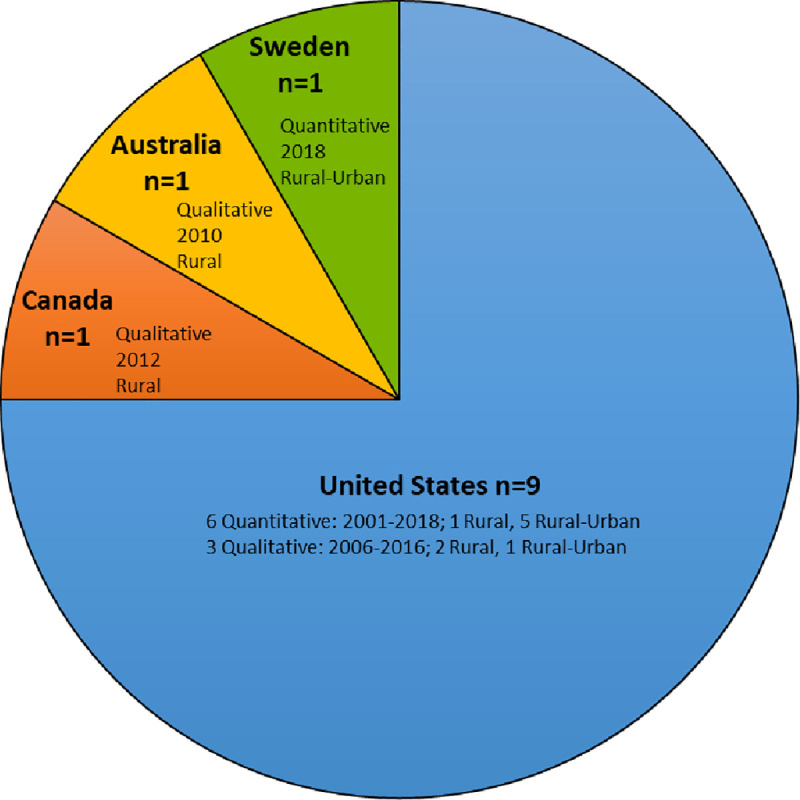
Characteristics of included peer-reviewed literature.

**Fig 3:**
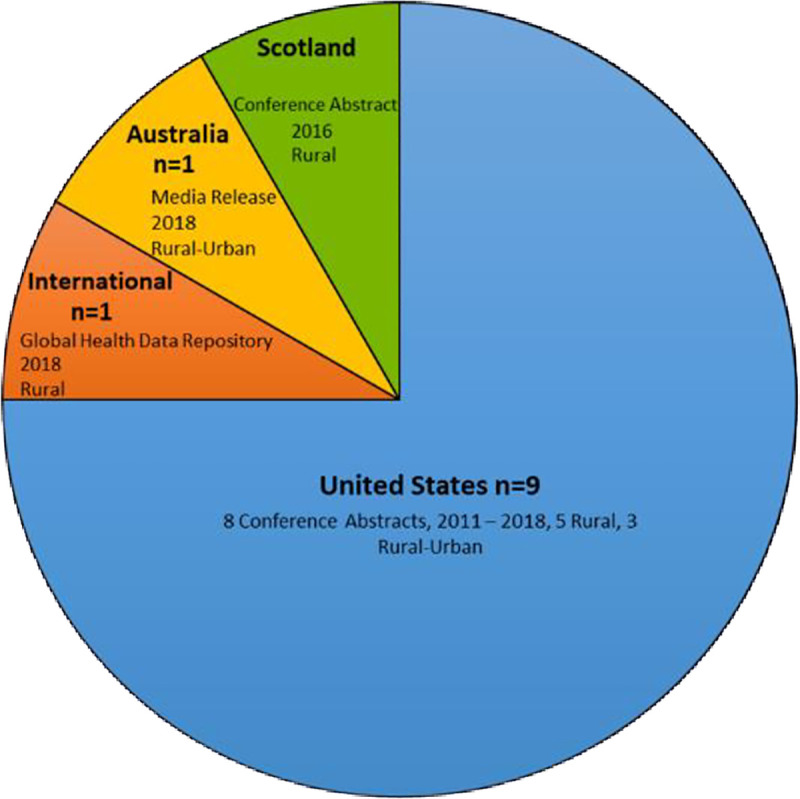
Characteristics of included grey literature.

Frequency distribution of the country of origin for the peer-reviewed literature and the grey literature (as well as additional numerical information regarding year, study design/document type, and whether rural or rural-urban) are illustrated graphically in Figs 2 and 3, respectively and charted data are presented in Tables 2 and 3, respectively. Analysis of the main findings identified five key overarching themes related to: 1) dementia knowledge, 2) services and supports, 3) decision-making, a person-centered approach and collaborative care, 4) artificial nutrition, hydration, and comfort care, and 5) quality of life and death. Themes are described in detail below and each of the five themes were mapped to the literature in Table 4.

**Table 2:** Charted data—peer-reviewed literature.

Author, Year, Country	Study Objective(s)	Methods, Sample	Rural or Rural-Urban, Definition of Rural	Main findings regarding rural/dementia/end-of-life	Reported Limitations, Conclusions, Recommendations^a^
**Quantitative (n = 7)**					
De Vleminck et al. [16] 2018 United States	To explore and describe characteristics of hospices that serve patients with dementia and compare hospice disenrollment patterns for patients with and without dementia, and assess both patient and hospice characteristics associated with hospice disenrollment	**Methodology:** Quantitative • **Retrospective cohort** ○ Medicare hospice claims data and National Hospice Survey (2008–2011) **Sample:** 7328 Medicare beneficiaries cared for in hospice with primary diagnosis of dementia • Rural/Suburban: 512 • Urban: 6816 **Sex:** 2078 male, 2520 female **Age:** 65+ years •2678 age 65–84 years • 4650 ≥ 85 years **Race:** 6348 White, 980 non-White	Rural/Suburban-Urban Definition of Rural: “Urban/Rural location measured as whether the hospice’s county had ≥ 1 million population”	• Hospices caring for people with dementia were more likely to: be for-profit, larger, provide 5+ years of care, and serve more patients from nursing homes • Hospice patients with dementia were more likely to disenroll after long stays (165+ days), particularly when served by smaller hospices, versus due to an acute event, compared to patients without dementia	**Limitations** • Data was collected several years prior to publication of this study published 2018 so “may not reflect current hospice market” • Data from National Hospice Survey and Medicare hospice claims do not include reasons for hospice disenrollment or its relationship with patient/family care preferences **Conclusions/Recommendations** • Results from this study point toward identifying what reasons/conditions are appropriate for hospice disenrollment of people with dementia and the importance of documenting these reasons • Sufficient guidance for hospices to identify patients appropriate for hospice admission is warranted, as is flexibility in hospice eligibility criteria for people with unpredictable disease trajectory like dementia
Lethin et al. [17] 2018 Sweden	To study and describe the care and services available to people with dementia and their informal caregivers, by further developing and testing a mapping system in the local context of Swedish municipalities, and to assess availability and utilization of care activities and professional providers’ educational level across categories of care (Screening, diagnostic procedures, and treatment; Outpatient care facilities; Institutional care and Palliative care)	**Methodology:** Quantitative • **Retrospective Cross-sectional** ○ Health care and social service systems data provided by dementia-related professional providers **Sample:** 9 municipalities (in 2 counties) • Rural: 3 • Urban: 6 with 200,641 residents (as of 2014, 1087 people had diagnosis of dementia) **Sex**: not reported **Age**: 0 to 65+ years • 21% 0–17 years • 58% 18–64 years • 21% ≥ 65 years **Race**: 24% “with foreign background”	Rural-urban Definition of Rural: Not reported; identifies three municipalities as representing rural areas	• Palliative care activities itemized in the mapping system remained unchanged and included hospice/institutional palliative care, hospice/palliative care at home, and advanced directive services • Mean available care activities (of 19) across all 9 municipalities was 13 • Availability and utilization of care activities along with professional’s education level were higher in general for screening/ diagnostics/treatment compared to outpatient care facilities, institutional care, and palliative care • Institutional care and residential homes were available across all 9 municipalities, with utilization differences and nursing homes available in over half of the municipalities, also with mixed utilization; most institutional care activities were well-utilized, primarily during late and end-of-life stages • Few dementia care or psychogeriatric units existed and where they did, they were utilized by “most or all” • Home and institutional palliative care was available in most municipalities across all stages of dementia but were underutilized (one municipality had zero palliative care available for anyone) • Advanced directive services were available to everyone in 2 municipalities but unavailable for most across the other 7 municipalities and where available, were underutilized except during end-of-life) • Institutional care specific to people with dementia was available in less than half of the municipalities for both rural and urban; where institutional end-of-life care was available, it was utilized by most (during late and end-of-life stages, utilization of institutional care and palliative care increased) • Professional carers specialized in dementia “were rare”; those who were typically worked in residential homes • Palliative care was provided by professional carers with a broad range of educational levels (post-secondary and Bachelor’s or Master’s degrees, some with specialization in dementia)	**Limitations** • Possible that availability and utilization response options lacked clarity • Data collected in 2015 may not be the same if collected at present time • Definitions/descriptions of care activities remain subjective • Quality of care activities “cannot be stated” but there were signs that education level played a role • No data regarding how responsibility for care and services was designated in real life (who is responsible for which care activities and who patients/families should turn to) • Reasons for differences between availability and utilization, and how care options are communicated to people who need them, should be examined further **Conclusions/Recommendations** • Mapping system is useful in allowing policy makers and professionals to see strengths/weaknesses in the health care and social services system and develop care and service systems that are equitable for people with dementia and caregivers during the entire disease course • Professional carers’ and service providers’ educational levels could help identify where the gaps are in the “chain of care” and what dementia-specific education is needed • Mapping system could allow professionals to be actively involved in communicating to people with dementia and their caregivers what care and services are available, over the entire course of the disease
Volandes, et al. [18] 2011 United States	To evaluate end-of-life preferences of older adult patients living in rural communities and assess if preferences are related to health literacy level	**Methodology:** Quantitative • **Randomized Controlled Trial** ○ Structured questionnaire interview ○ Structured interview ○ Verbal description of advanced dementia ○ Goals of care video decision aid (Intervention group only) **Sample:** 76 older adults cared for at a rural primary care clinic, without moderate or severe cognitive impairment or diagnosis of dementia • Rural: 76 • Urban: 0 **Control Group:** 43 **Sex:** 13 male, 30 female **Age:** mean (SD) 75 years (6 years) **Race:** 23 White, 20 Black or African American **Marital status:** • 14 married • 24 widowed • 4 divorced • 1 never married **Education:** • 6 elementary • 12 some high school • 15 high school graduate • 5 some college • 2 college graduate • 3 post-graduate or professional **Intervention Group:** 33 **Sex:** 14 male, 19 female **Age:** mean (SD) 73 years (6 years) **Race:** 17 White, 16 Black or African American **Marital status:** • 16 married • 10 widowed • 6 divorced • 1 never married **Education:** • 5 elementary • 10 some high school • 6 high school graduate • 5 some college • 3 college graduate • 4 post-graduate or professional	Rural Definition of Rural: None provided but states the recruited participants were “cared for at a primary care clinic located in rural Greensburg, Louisiana, a federally designated Health Professional Shortage Area” and that “the clinic is reflective of the village of Greensburg”	• Health literacy levels Control group: <6^th^ grade (n = 12), 7-8^th^ grades (n = 10), ≥9^th^ grade (n = 21) Intervention group: <6^th^ grade (n = 12), 7-8^th^ grades (n = 6), ≥9^th^ grade (n = 15) • Comparisons were drawn regarding preferences for life-prolonging care (CPR, etc.), limited care (hospitalization but no CPR, etc.), and comfort care (symptom relief) • Participants with higher health literacy levels were more likely to choose comfort care • Participants who viewed the decision-aid video (the intervention group) were more likely to prefer comfort care (91%) versus the control group (72%) • Limited care was preferred by 9% of the intervention group versus 5% of the control group • None of the intervention group preferred life-prolonging care versus 16% of the control group • In general, preference for comfort care across both the control group and the intervention group were associated with: ○ being white (4x more likely), ○ being female (3.6x more likely), ○ randomization to intervention group (3.9x more likely), ○ greater level of health literacy (p = 0.003), especially >9th grade level (12x more likely) compared to 6th grade level • All of the intervention group found it [video] to be helpful and would recommend to other older adults	**Limitations** • People outside of the primary health care setting were not evaluated (study participants were enrolled at their primary care clinic which means the study sample already had access to health services) • Potential bias (interviewer was not blinded to randomization allocation) • Findings may not be generalizable (small study with 76 participants from one rural area in Louisiana) • Roles of health literacy, gender, and race could not be examined separately (low statistical power) • No variation in video illustration of patient and clinician race/ethnicity/ gender, or clinical setting (video portrayed only white patients with an African American clinician/narrator) **Conclusions/Recommendations** • Future studies should examine the effects of videos that include different patient/clinician aspects in a variety of settings and regarding other diseases
Miller et al. [19] 2010 United States	to examine differences and changes over time in hospice use (such as enrollment and length of stay) among United States nursing home residents who are dying with advanced and mild-to-moderately severe dementia	**Methodology:** Quantitative • **Retrospective longitudinal** (1999–2006) • **Retrospective Cross-sectional** (2006) ○ Center for Medicare and Medicaid Services (CMS) Nursing Home resident assessment data minimum data set (MDS) 1999–2006 for the 50 US states and the DC, matched to Medicare Part A claims data (ie, for hospice, hospital, home health, outpatient, and skilled nursing facility care and to Medicare enrollment data • **Longitudinal analyses** used proportions and means for hospice enrollment and lengths of stay over time • **Cross-sectional analyses** used proportions and means were to compare decedent characteristics with advanced versus mild-to-moderate and within each group, who did and did not access hospice in the nursing home; also compared rates of hospice enrollment and lengths of hospice stay across the 50 US states and DC. **Sample:** 293,473 Medicare/Medicaid-certified nursing home decedents (with complete MDS data) with dementia (specific numbers regarding rural and/or urban location not provided) **Advanced dementia n = 85,942** **Sex:** 70.4% female **Mean age at death:** 85 years **Race:** • 86% non-Hispanic white • 9.6% non-Hispanic black • 2.9% Hispanic • 1.3% Other **Marital status:** • 2.7% married • 44.6% widowed • 13.6% single **Education:** • 35.6% less than high school • 39.2% high school • 21.2% some college • 4% no data Mild to moderately severe dementia n = 117,531 **Sex:** 65.1% female **Mean age at death:** 86 years **Race:** • 89.4% non-Hispanic white • 6.9% non-Hispanic black • 2.3% Hispanic • 1.2% Other **Marital status:** • 24.5% married • 60.9% widowed • 14.5% single **Education:** • 34.9% less than high school • 40.1% high school • 22% some college • 2.8% no data	Rural-urban Definition of rural: None provided but identifies Alaska, Wyoming, Hawaii, and Vermont as” more rural states”	**Longitudinal (1999–2006):** • Hospice access/use change for NH decedents with advanced dementia tripled between 1999 (14.5%) and 2006 (42.5%) and almost tripled for decedents with mild-to-moderately severe dementia (13.2%-1999 to 37.9%-2006) • Increased access/use was associated with longer mean stays, especially for those with advanced dementia (from 46.1 days in 1999 to 118.2 days in 2006), compared to mild-to-moderately severe dementia (from 38.7 days to 79.5 days in 2006) **Cross-sectional (2006)** **Compared to the mild-to-moderately severe groups, the advanced dementia decedents were:** • More often younger, female, had at least a high school education, a stroke diagnoses, do-not-hospitalize (DNH) or do-not-resuscitate (DNR) orders, and long (>90 days) nursing home stays • Less often non-Hispanic white, less likely to have had diagnoses of arteriosclerotic heart disease (ASHD), cancer, chronic obstructive pulmonary disease (COPD), congestive heart failure (CHF), other cardiovascular disease (CVD), and renal failure • Had longer mean hospice stays in 2006 due to having fewer short hospice stays and more longer stays, compared to the mild-to moderate group • Were overall more functionally and cognitively impaired • With comorbid stroke diagnoses, were less likely to access hospice (those with mild-to-moderate dementia and stroke diagnoses were slightly more likely) **Across both advanced and mild-to-moderate groups:** • Those with nursing home hospice use (compared to no nursing home hospice use) were more often female, non-Hispanic white, and had higher levels of education, cancer diagnoses, other CVD, renal failure, DNH or DNR orders, and long nursing home stays • Fewer had ASHD, COPD, CHF, or were enrolled in health maintenance organizations (HMOs) • Those with a comorbid cancer or other CVD were more likely to access hospice compared to those without those diseases **State variation in hospice use for decedents with:** Advanced dementia: • Lowest rates of hospice use among “more rural” states of Alaska (2.7%), Wyoming (5.4%), Hawaii (9.9%), Vermont (10.9%) • Highest hospice use among Texas (59.2%), Kansas (60.6%), Iowa (62.2%), Oklahoma (71.7%) • **Mild-to-moderate dementia:** • Lower rates of hospice use compared to those with advanced dementia in 46 of 50 US states and in DC (proportions not provided) • In almost all states, median hospice stays much lower than the advanced dementia group • **Across advanced dementia and mild-to-to-moderate dementia groups, hospice lengths of stay varied:** • 3 states (Oklahoma, 155 days; South Carolina, 73 days; Alabama, 71 days) had significantly longer mean lengths of stay • In Oklahoma, 46.6% of nursing home hospice decedents had stays greater than 180 days, whereas these proportions were less so South Carolina (33.2%) and in Alabama (34.3%)	**Limitations** • Diagnosis of dementia and grouping into advanced and mild-to-moderately severe were determined indirectly (using secondary data in the MDS and Medicare claims) • Although this research describes hospice use by nursing home decedents with dementia, there were no secondary data regarding certain potential factors related to hospice referral (e.g., decision making) • **Conclusions/Recommendations** • People with dementia dying in nursing homes have increased access to Medicare hospice care, but considerable variation to access exists throughout the United States • Mean lengths of hospice stay for people with dementia have increased but high proportions of decedents with advanced dementia had very long stays and high proportions with mild-to-moderate dementia had very short stays • Recommended that providers continue documentation of their reasons for referral to hospice based on a predicted 6-month survival
Mitchell et al. [20] 2007 United States	to describe hospice recipients who died with dementia, and their family’s assessment of hospice care; to compare and the experience of dementia decedents with that of older persons with “other common terminal illnesses”	**Methodology: Quantitative** • **Retrospective cohort** ○ Family Evaluation of Hospice Care (FEHC) survey data (from across 796 hospices across United States, where for this study, 23 were excluded due to mean annual survey response rate being <20%), excluding decedents with AIDS or unknown primary reason for hospice referral and those under aged 65 years upon death **Sample:** 77,123 bereaved family members of hospice recipients who died with primary illness of dementia (n = 8686), cancer (n = 35,693), or other chronic diseases (32,744) **Primary Illness of Dementia (n = 8686)** Hospice Recipient: • **Age:** 57.2% over 85 years of age • **Sex:** 68.9% female • **Race:** 94% White • **Education:** ≥ High school: 69.6% Bereaved Family Member: • **Age:** ○ 14–44 years: 5.2% ○ 45–54 years: 19.3% ○ 55–64 years: 32.8% ○ 65–74 years: 20.5% ○ ≥ 75 years: 22.3% • **Sex:** 69.7% female • **Race:** 94.4% White • **Education:** ≥ High school: 93.7% Hospice: • Rural: 9.8% **Primary Illness of Cancer (n = 35,693)** Hospice Recipient • **Age:** 23.1% over 85 years of age • **Sex:** 49.3% female • **Race:** 93% White • **Education:** ≥ High school: 73.1% Bereaved Family Member: • **Age:** ○ 14–44 years: 8.9% ○ 45–54 years: 20.4% ○ 55–64 years: 22.9% ○ 65–74 years: 25.6% ○ ≥ 75 years: 22.1% • **Sex:** 73.4% female • **Race:** 93.5% White • **Education:** ≥ High school: 91% Hospice: • Rural: 13.5% **Primary Illness—Other chronic disease:** Hospice recipient: • **Age:** 53.8% over 85 years of age • **Sex:** 58.8% female • **Race:** 94% White • **Education:** ≥ High school: 66.5% Bereaved family member: • **Age:** ○ 14–44 years: 6.2% ○ 45–54 years: 18.8% ○ 55–64 years: 31.4% ○ 65–74 years: 22.9% ○ ≥ 75 years: 20.7% • **Sex:** 74.2% female • **Race:** 94.6% White • **Education:** ≥ High school: 91.8% Hospice: • Rural: 13%	Rural-urban Definition of rural: “urban/rural designation” determined using “hospice zip codes with the 2005 Area Resource File”	**Compared to characteristics of hospice recipients and programs described in a nationwide 2006 report** from the Medicare Payment Advisory Commission APAC report, in this study sample: • More hospice recipients were <65 years of age (15.1% vs. 5.1%); but authors excluded this age group from their analyses • All hospice recipients in this study were deceased whereas only 82.7% of those in the MedPAC sample had died • Otherwise, demographic features of the hospice programs and recipients in this study were comparable to the MedPAC sample **Hospice Recipient Characteristics** • Compared to the cancer group, the dementia group had significantly more decedents >85 years of age (57.2% versus 23.1%) • Those with dementia were more likely female (68.9%) compared to those dying with cancer (49.3%) and other chronic diseases (58.8%) • Significantly more of those with a primary diagnosis of dementia (13.7%) had a length of stay >180 days compared to those with cancer (5.3%) and chronic diseases (8.5%). • No meaningful differences existed regarding race and education among decedents by disease groups (i.e., >5.0%), with most being White and having at least a high school education **Bereaved Family Member Characteristics** • Similar characteristics of survey respondents (bereaved family members) existed among all three decedent groups regarding age, gender, education, and race • Similar respondent-hospice recipient relationships existed between dementia and chronic disease (CD) groups (just over half were children of the decedents, and a quarter were spouses). In contrast, survey respondents in the cancer group were more likely spouses (45.2%) than children (38.2%) • Respondents’ reports of the timing of hospice referral was similar across all groups, where most felt the timing was about right (88.0%), 1.3% felt it was too early, and 10.8% felt it happened too late **Hospice Characteristics** • Dementia decedents were more likely to receive care from for-profit (22.9%) and freestanding (69.9%) hospices compared to those dying with cancer (for profit, 10.8%; freestanding, 60.1%) and chronic diseases (for profit, 14.6%; freestanding 62.3%); in contrast, hospice recipients with cancer and CDs were more likely in hospital-based hospices • Those dying with dementia were more likely being cared for in hospices where “the volume of dementia decedents exceeded 10%” • 12.9% of hospices were rural • Hospices’ average daily census, location (i.e., urban vs. rural), and mean annual survey response rates were similar among all three disease groups **Hospice Care** • Most respondents rated overall hospice care as excellent regardless of decedents’ primary diagnoses (dementia, 73.2%; cancer, 77.9%; and CDs, 75.6%). • Similar numbers of respondents in all groups reported at least one issue with care coordination (17.5%), information received from providers about patients’ overall condition (20.1%), information received about treatment of patients’ symptoms (12.2%), and provision of emotional support to the family (29.8%) • Unmet patient needs for symptom treatment were comparable in all decedent groups (pain, 5.5%; dyspnea, 4.7%, anxiety/sadness, 9.3%)	**Limitations** • Not all hospices in the United States use the Family Evaluation of Hospice Care (FEHC) survey • Survey response rates differed among programs (free standing, hospital, home-health agency, other) • Survey items had different proportions of missing values • Survey data did not include site of hospice care (e.g. nursing home, home, etc.) **Conclusions/Recommendations** • Hospice services for recipients with dementia appeared to be beneficial however, only a small portion accessed hospice care • Most commonly concerning areas identified by bereaved family members included were a need for: “better emotional support, coordination of care, and information about what to expect during the dying process”
Gessert, Haller et al. [21] 2006 United States	To identify factors related to the use of selected medical services near the end of life for residents of rural and urban [United States] nursing homes with severe dementia	**Methodology:** Quantitative • **Retrospective cohort study** ○ Minimum Data Set (MDS) report (containing the Centers for Medicare and Medicaid Services administrative data) for residents of nursing homes in rural and urban counties within Minnesota and Texas between January 1, 2000 and March 31, 2002 with “severe and chronic cognitive impairment” who died during 2000 to 2001 (excluding resident under age 67 years, HMO enrolment or hospice last 2 years prior to death, and those in nursing homes rated neither very rural nor very urban) **Sample:** 3710 (from 1016 nursing homes) • **Rural (n = 1886):** • **Sex:** 453 male, 1433 female • **Age:** 87.8 years (mean) • **Race:** 92.5% White, 17.5% Nonwhite • **Dementia:** n = 1272 with dementia (565 Alzheimer’s disease, 887 other dementia) • **Urban (n = 1824):** • **Sex:** 424 male, 1400 female • **Age:** 86.7 years (mean) • **Race:** 79.1% White, 20.9% Nonwhite • **Dementia:** n = 1360 (521 Alzheimer’s disease, 839 other dementia)	Rural-urban Definition of Rural: based on the U.S. Department of Agriculture 1993 metro—non-metro continuum county codes where urban counties were metropolitan areas, midsize counties included urban populations ≥20,000 or at least 2500 and “adjoined a metropolitan area”, and rural counties were “those with smaller populations”	• Most residents had dementia (1272/1886 rural and 1360/1824 urban) • During the last 90 days of life rural nursing home residents were more likely than urban residents to have do-not-resuscitate orders and/or living wills and Medicaid coverage • During the last 90 days of life rural nursing home residents were less likely than urban residents to be hospitalized for less than 10 days, use a feeding tube, be admitted to intensive care, or have a stroke • Overall, more use of all services was associated with being nonwhite and having had a stroke; specifically, nonwhite nursing home residents were more likely to use all services in urban nursing homes, more likely to have used feeding tubes; urban residents were less likely to be hospitalized than rural but more likely to be hospitalized for longer or in intensive care than those in rural nursing homes • Having Medicaid coverage was associated with less hospitalization • Advance directives were associated with less likely hospital admission and intensive care across both rural and urban nursing home residents • Most (over 75%) of the study population was from Texas and more than half of these resided in urban nursing homes (versus just over 70% of Minnesota nursing homes residents were rural) • Most (64%) rural nursing homes were smaller (<100 beds) and less likely than urban (82% versus 65%) to be for-profit	**Limitations** • Limitations of administrative data [none explicitly specified] • Exclusion of nursing home residents covered by hospice or HMO in last 2 years of life (“exclusion of hospice beneficiaries affected a larger proportion of urban (38%) than rural (13%) nursing home residents”) • Facility-level factors were not included in analysis due to small numbers of residents in each facility • Study design did not allow for examination of regional differences “per se” • **Conclusions/Recommendations** • “Rural nursing home residence is associated with lower likelihood of use of the most-intensive medical services at the end of life” • Facility level factors and regional differences should be examined in future research
Gessert & Calkins [22] 2001 United States	To examine “the use of feeding tubes in a population of nursing home residents with severe and irreversible dementia, with particular attention to rural-urban variation in feeding tube use”	**Methodology:** Quantitative • **Retrospective cross-sectional** ○ Minimum Data Set (MDS) report (containing the Centers for Medicare and Medicaid Services administrative data) for residents of nursing homes in rural, midsize, and urban counties in Kansas between January 1, 1994 and June 30, 1998, with “severe and irreversible dementia” (excluding residents under 65 years of age, those in a coma, and where “MDS report of interest did not include key demographic data”) **Sample:** 4847 nursing home residents (506 of which had feeding tubes) (36.4% rural, 27.1% midsize, 36.5% urban) **Rural (n = 1762):** • **Sex:** 79.4% female • **Age:** 60.8% age 86+ years • **Race:** 2% nonwhite **Midsize (n = 1315):** • **Sex:** 78.7% female • **Age:** 58.5% age 86+ years • **Race:** 3.4% nonwhite **Urban (n = 1770):** • **Sex:** 78.7% female • **Age:** 52.2% age 86+ years • **Race:** 11.8% nonwhite	Rural-urban Definition of Rural: based on the U.S. Department of Agriculture 1993 metro—non-metro continuum county codes where urban counties were metropolitan areas, midsize counties included urban populations ≥20,000 or at least 2500 and “adjoined a metropolitan area”, and rural counties were “those with smaller populations”	• Nursing home residents in rural and midsize counties were more likely older, white, and not eligible for Medicaid, compared to those in urban counties, but with similar clinical characteristics among all three groups • Feeding tube use was associated with nursing home residents in urban counties (19.3%) compared to those in midsize (8%) and rural (6.4%) counties • Overall, feeding tube use was significantly higher in urban counties for most subpopulations (men, women, whites, nonwhites, and those eligible and ineligible for Medicaid) • Feeding tube use was greatest among urban nursing home residents among clinical subpopulations such as Alzheimer’s disease or stroke • Feeding tube use was significantly associated with: ○ Geographic location (rural less than urban) ○ Nonwhite race (versus white) ○ Being male ○ Underlying clinical condition (stroke versus AD) ○ Greater dependency (ADL) ○ Absence of a living will • Strongest overall association with feeding tube use was swallowing problems and there were more swallowing problems among urban residents versus midsize and rural • Even for residents without swallowing problems (n = 2,769), tubes were more frequently used among urban (5.9%) versus midsize (3.0%) or rural (2.6%) (p < .001) • Similarly, even for the residents without chewing problems (n = 1,555), feeding tubes were more used among urban (31.4%) versus midsize (14.6%) or rural (13.6%) (p < .001)	**Limitations** • Retrospective descriptive design using existing administrative data to identify associations (vs cause-effect • Low generalizability—data was from only one state (Kansas) (largely rural, white population, and “silent on end-of-life care and patient feeding”) **Conclusions/Recommendations** • Sparse literature exists on differences in current end-of-life practices between different communities • Important to identify and study “best practices” in different settings, including rural communities • Study findings demonstrate that alternatives to feeding tubes (such as “assisted oral feeding” are accepted medical practice in severely demented patients, in that 88.4 percent of the study population did not have feeding tubes (even though inclusion criteria included all study subjects be ‘eating-dependent’ • Further investigation regarding why differences of feeding tube use occurs using qualitative methods is warranted, in particular to examine rural-urban differences
**Qualitative (n = 5)**					
Smith et al. [23] 2016 United States	To evaluate the perspectives of home healthcare nurses’ regarding the use of artificial nutrition and hydration for people with late stage dementia and whether their perceptions and beliefs influence them while providing care for patients with severe dementia and their families	**Methodology:** Qualitative • Focus Groups ○ Semi-structured interview questions ○ 5 open-ended interview questions **Sample:** 17 home healthcare nurses in “rural southern United States” (North Carolina), recruited via convenience sampling over 4 months, who worked in a Medicare-certified home healthcare agency, worked in the field, saw patients in-home or residential care facility, and provided direct care to at least one patient with late-stage dementia during their time as a home healthcare nurse • Rural: 17 (all) • Urban: None **Sex:** 3 male, 14 female **Age:** 65+ years • 2678 age 65–84 years • 4650 ≥ 85 years **Race:** 17 (all) White **Education:** • 71% Associate degree • 53% had 20+ years of experience as a registered nurse 58% had less than 15 years of experience as home healthcare nurse	Rural Definition of Rural: None provided but states the study participants were from “a rural area in the southern United States”	• Most nurses expressed a focus on patient and/or family comfort where artificial nutrition and hydration were often provided as “comfort measures” for both • Some nurses identified that artificial nutrition and hydration as a complex issue which can make end-of-life decision-making difficult for the family • Many nurses expressed feeling “helpless in knowing how to care for people with dementia” and expressed the belief that while artificial nutrition and hydration can be comforting to the family, it can also increase suffering for the patient • Examples of related suffering were described by nurses where tube insertion is “a painful invasive procedure” which at times requires patient restraint to avoid pulling out the tube; risk of infection or aspiration; and increased fluid which can often cause “discomfort, nausea and vomiting, and diarrhea” • Many nurses stated that even though in general they often see artificial nutrition and hydration as futile and would prefer not to see patients suffer in this way, they always discuss these options with the patients’ families and discuss the patients’ wishes • Many nurses believed artificial nutrition and hydration extended a patient’s life and although that this could be viewed as a “benefit to the family but not to the patient” in terms of reducing the patients’ quality of life and prolonging suffering and also in terms of providing a sense of “false hope” to the family • Symbols of such suffering were conveyed by nurses, primarily within comments related to the physicality of the disease, and the difficulty of assessing the degree of patients’ pain and suffering when they have lost the capability of “purposive language”	**Limitations** • None reported **Conclusions/Recommendations** • Perceptions of nurses in this study exemplified the importance of their beliefs are influential on the care provided to patients and families who are considering artificial nutrition and hydration during late-stage dementia • Importance of gaining and using evidence-based knowledge/information of decisions regarding artificial nutrition and hydration at end of life so that families are provided with the necessary information they need to fully understand all aspects related to the decision of whether or not to initiate artificial nutrition and hydration for their family members with late stage dementia • Future research should be aimed at establishing a more concise definition and measure of suffering in people with late stage dementia
Forbes et al. [24] 2012 Canada	To explore the information needs regarding dementia, how these needs change as the dementia advances, and how the knowledge is used, among healthcare providers, caregivers, and people with dementia, to allow for the most effective use of the information	**Methodology:** Qualitative • In-person interviews (at study entry, 6 months, and 1-year) ○ Guided interview questions **Sample:** 9 ‘dementia care networks’ consisting of 5 rural persons with dementia, rural 14 care partners, and 14 healthcare practitioners, recruited via convenience (through study collaborators) followed by purposive sampling • Rural: 17 (all) • Urban: None **Persons with dementia (n = 5)** **Sex:** 3 male, 2 female **Age:** mean (SD) 77.40 years (11.67) (range 63 to 95 years of age) **Marital status:** • 3 married, 2 single **Education:** • 1 completed post-secondary • 2 secondary or trade school • 2 primary school or less **Employment status:** all retired/not employed **Community population:** • <1000: 1 • 1000–4999: 3 • 5000–10000: 1 **Mean Kilometers from urban center:** 26.3 (+/-12) (range 21.9 to 29.1) **Care partners (n = 14)** **Relationship with person with dementia:** • 6 spouses • 5 children • 2 grandchildren • 1 nephew **Sex:** 3 male, 11 female **Age:** mean (SD) 60.31 years (15.74) (range 39 to 91 years of age) **Marital status:** • 11 married, 3 single **Education:** • 1 some post-secondary • 6 completed post-secondary • 6 secondary or trade school • 1 primary school or less **Employment status:** • 6 full-time employed • 8 retired/not employed **Community population:** • <1000: 1 • 1000–4999: 9 • 5000–10000: 1 • >10000: 3 **Mean Kilometers from urban center:** 33.4 (+/-10.79) (range 21.9 to 61.1) **Community-based healthcare practitioners (n = 14)** **Role:** • 3 case managers • 1 personal support worker • 2 social workers • 3 physicians • 3 Victoria Order of Nurses coordinators **Sex:** 9 male, 5 female **Age:** mean (SD) 45.71 years (13.15) (range 24 to 63 years of age) **Marital status:** Not provided **Education:** • 4 graduate/professional • 8 completed post-secondary • 1 secondary or trade school • 1 primary school or less **Employment status:** • 13 full-time employed • 1 part-time employed **Community population:** Not provided • **Mean Kilometers from urban center:** Not provided	Rural Definition of Rural: based on Statistics Canada, as “living outside a major urban centre of more than 10 000 population”.	• Only 5/9 persons with dementia from the dementia care networks were willing or able to participate in this study • All persons with dementia were diagnosed with Alzheimer’s disease • Most (n = 4) persons with dementia were living with dementia for 5 years or less and n = 1 for more than 5 years • Self-reported health status of persons with dementia varied equally across a range from poor to excellent • Self-reported health status of care partners varied across a range from poor to excellent with most (10/14) reporting good or excellent, 3 fair, and 1 poor Healthcare practitioners (HCPs): • In general, identified end-of-life care discussions with care partners as important to provide information about options for treatment, support, and expectations but admitted discussion was often delayed or avoided due to difficulties and reservations of broaching the issue Family members/care partners: • Many avoided a discussion about end-of-life care and expressed not wanting to look too far ahead however, some expressed gratitude for having the discussion and felt more prepared moving forward • Many reported accessing information related to end-of-life care services/interventions and supports from other family members that were HCPs, their physicians and/or health centers, community care organizations/facilities, support groups, and the internet • In general, reported end-of-life decisions were often made in consultation with other family members and the physician of the person with dementia	**Limitations** • Persons with dementia and their care partners who were not receiving formal healthcare services may have different experiences than those who were receiving such services however, they were not included in this study **Conclusions/Recommendations** • This study identified the importance of *early* discussion between HCPs, persons with dementia, and care partners/family members to informatively plan for the future regarding the ‘rural dementia care journey’, available options for care, and to provide the chance for people with dementia to communicate their wishes
Lindsay et al. [25] 2010 Australia	To “describe a community collaborative model of care for end-stage dementia” by presenting a case study that exemplifies the experience of a rural couple, one with end-stage dementia and the other as caregiver, who wanted the chance to die at home, and how a local dementia service was able to recognize and meet the couple’s support needs. Also, to examine how case studies “can inform future practice initiatives”	**Methodology:** Qualitative • Case Study **Sample:** 1 individual with dementia and 1 spousal caregiver • Rural: both • Urban: None **Sex:** 1 male, 1 female **Age:** Not provided **Marital status:** • Married **Education:** • Male: some college • Female: not provided **Employment:** • Male: retired farmer • Female: homemaker	Rural Definition of Rural: None	• Case study presentation (narrative description) of a farmer with dementia and his spousal caregiver who wished to remain at home on the farm until his death and were able to do so through implementation of a community collaborative model for end-stage dementia care and adoption of a person-centered, case-management approach • After an initial geriatrician referral, for one year (until one week before her husband’s death) the couple had attended a multidisciplinary community dementia care service (1 psychologist, 4 nurses, 1 diversional therapist) that provided up to 4 hours of day care with meaningful activities and behavioural interaction along with caregiver support and education • Caregiver spouse had regular discussion with a case manager, as her husband’s dementia progressed, to identify and address their changing needs • Case manager contacted and involved in the couple’s care General Practitioners along with “other professionals from community care teams” to ensure needs were met • Needs changed over time throughout disease progression to final stages of dementia (e.g., initial needs identified and met were education and support; end-of-life care included palliative care in the home along with grief and bereavement counselling for spousal carer after death of her husband) • “residential respite care” was available but both carer and spouse with dementia expressed anxiety and concern; specifically, the carer was concerned about giving up her role as primary caregiver and worried whether respite facility would meet husband’s needs; on occasion when respite care was utilized, her husband responded negatively (‘get me out of here’); both expressed their desire to stay together at home on their farm • Case manager was able to identify to the spousal carer when her husband was nearing the end-of-life stage at which point options for care were discussed and support was offered to meet their desire of allowing him to die at home • One week before his death the case manager referred to the palliative home care team (support included pain relief, medication for agitation, and advice to the family re: oral and pressure area care); the case manager stayed in contact with the spousal carer throughout this process which provided continuous contact and reassurance from the same (familiar) person • Spousal caregiver expressed that her husband being able die at home gave them a sense of relief and allowed herself and other family members more opportunity for spiritual reflection • Initially and weeks after her husband died, the spousal carer expressed gratitude and appreciation for the support and assistance that allowed her husband to die at home—- so much so that she reflected on it as ‘a positive experience’ and ‘wouldn’t change a thing’	**Limitations** • None reported **Conclusions/Recommendations** • Education and practical support by health professionals can be empowering and allow for “individual and informed choices” in spite of rural complexities (e.g., geographical barriers; care professional doubt whether rural persons with dementia can be adequately supported to remain in their home until death) • Flexibility of a person-centered case management approach is much needed for families and for referrals to the correct care professionals • “The assumption that all people with end-stage dementia will require nursing home placement is incorrect”; “the experience of [this couple] demonstrates that dying at home can be a positive experience” • Future research and support for rural people with end stage of dementia and their caregivers is a priority, particularly for those people who choose to die at home, to enable them to do so
Modi et al. [26] 2010 United States	To better understand community view about feeding tube use for patients with end-stage dementia and explore any differences in the views of African-Americans versus Caucasians	**Methodology:** Qualitative • Focus Groups ○ Guided interview questions **Sample:** 28 community members • Rural: “largely rural” • Urban: not reported **African American (n = 11)** **Sex:** 3 male, 8 female **Age:** Mean (SD) 58.6 years (7.2 years) **Marital Status:** 5/11 married **Education:** • 1/10 college degree or greater • 5/10 beyond high school **Employment:** • 2/9 employed outside the home **Yearly household income:** • 2/9 above $35K • Median $25K-$35K **Health Insurance:** 11/11 **Caucasian (n = 17)** **Sex:** 6 male, 11 female **Age:** mean (SD) 68.5 years (7.6 years) **Marital Status:** 11/17 married **Education:** • 8/17 college degree or greater • 11/17 beyond high school **Employment:** • 4/17 employed outside the home **Yearly household income:** • 8/13 above $35K • Median $35K-$45K **Health Insurance:** 16/17	Rural Definition of Rural: None provided but states “largely drawn from rural communities”	• 77% of African Americans and 41% of Caucasians had cared for an elderly relative or friend at some point • 33% of African Americans and 11% of Caucasians were currently caring for an elderly relative or friend • 37% of African Americans and 94% of Caucasians expected to care for an elderly relative or friend in the next 5 years • All African Americans and 94% of Caucasians knew what a feeding tube was • 88% of African Americans and 64% of Caucasians had known someone who had had a feeding tube • Views of end-of-life tube feeding for people with dementia were similar overall among African American and Caucasians where both expressed concerns but were generally supportive of its use; however, African American participants also spoke about the negative historical injustices, particularly regarding the “use of African Americans as ‘experiments’ in health care” **In general (across both African American and Caucasian participants):** • Emphasis on the “the importance of food as a symbol of caring” and families were inclined to be concerned with staying committed to the “personal fidelity” to caring for the person with dementia “and the symbolic role of feeding in fulfilling that fidelity”; many identified providing food to someone who is sick is ‘human nature’ and to deny that is ‘going against the grain’ • Expressed wanting to ‘do the right thing’ and honor wishes (if known) of the person with dementia; • Many mentioned the “value of planning ahead” (e.g. advanced directives) however, some voiced concern about “medical personnel following directives” • Regarding medical decision-making, some participants expressed the importance of their own wants and beliefs, some felt they were not provided with understandable, adequate information and alternatives (such as a second opinion) • Some mentioned feeling that tube feeding was worse than death and prolonged suffering, and valued a natural death; others acknowledged the importance of tube feeding to prevent starving, to provide comfortability, to extend life until all family members are present • Some identified “age and cognitive status” as key factors to consider in decisions about feeding tubes • Many expressed religious views of life and death being up to God as well as a “belief that health professionals can override family and individual decisions” due to the Hippocratic Oath or hospitals fear of being sued if health professionals do not place a feeding tube when ‘needed’	**Limitations** • Findings may reflect influence of geographical and cultural differences as well as age (50–75 years) specific to study population • Small sample size, focus group moderator bias **Conclusions/Recommendations** • None reported
Gessert, Elliott, et al. [27] 2006 United States	To understand and describe differences between rural and urban families who have a family member with dementia residing in a nursing home	**Methodology:** Qualitative • Focus Groups ○ Guided interview questions **Sample:** 38 family members (of 35 nursing home residents with “severe cognitive impairment”, from 8 nursing homes [5 rural, 3 urban]in each of the 11 urban and 65 rural counties) who lived within 50 miles of the nursing home, respectively **Rural (n = 26):** **Sex:** 5 male, 21 female **Age:** 61 years (mean) **Race:** White **Relationship with nursing home resident:** 77% child or spouse • Family member in nursing home (n = 26): ○ 6 males, 17 females ○ Mean age: 86 years ○ Mean time in residence: 3.4 years **Urban (n = 12):** **Sex:** 1 male, 11 female **Age:** 63 years (mean) **Race:** 91% White **Relationship with nursing home resident:** 83% child or spouse • Family member in nursing home (n = 12): ○ 3 males, 9 females ○ Mean age: 91 years Mean time in residence: 4.6 years	Rural-urban Definition of Rural: based on the U.S. Department of Agriculture 1993 metro—non-metro continuum county codes	• **Both rural and urban** participants similarly reported being “active in religion” (rural 76% and urban 82%) • Both rural and urban participants considered themselves advocates for their love one’s care • Most rural participants reported unconditional acceptance of death as a natural process and with few desires other than hoping the process was both quick and peaceful; most also preferred death to happen early and did not wish for life-sustaining interventions merely to delay death • In contrast, variability existed among urban participants regarding attitudes toward death: ○ many never made any reference to death whatsoever; most who did expressed desire for family member to have a “peaceful death” but with a number of conditions, especially when “death would be accepted” ○ many favored more aggressive treatment and were open to life-sustaining interventions for their loved one compared to rural participants • many strongly opposed any acceptance of nearing death and most others who were more accepting of death appeared to be hesitant and unsure, often more accepting of a “distant death”; several reported being displeased with clinicians who encouraged palliative or hospice care	**Limitations** • other than having required family members live within 50 miles of their loved ones’ nursing homes, no rural/urban background screening was completed •focus group participants were self-selected and no demographics were collected for the individuals who turned down focus group participation • urban sample had more potential for selection bias (fewer families participated) • initial portion of each focus group weighed heavily with “discussions of how dementia had changed the lives of the family caregivers” therefore family members most involved in their loved one’s care and those experiencing the most burden might have been overrepresented • study population was primarily of White race (typical of the region) therefore the findings may not be applicable to more racially varied populations **Conclusions/Recommendations** • Findings from this study indicate that views of death for rural dwellers might be less complicated (i.e. more accepting of death) compared to those living in urban settings; however, these views may also be reflective of the relatively smaller and more homogenous community (e.g., more shared experiences, similar cultures/beliefs, etc.) compared to larger more diverse urban populations • Future research should explore not only geographical influences and differences (i.e. rural and urban) but also factors such as religion, ethnicity, and language

^a^Limitations, conclusions, and recommendations listed in this table were those reported in the original articles.

**Table 3:** Charted data—Grey literature.

RefID Author, Year, Country	Document Type	Document Title and/or Objective	Methods and/or Topics Addressed	Rural or Rural-Urban,Definition of Rural	Main findings/information regarding rural/dementia/end-of-life
Franklin et al. [28] 2018 United States	Conference abstract	Being in Two Places at Once: Utilizing Clinical Video Telemedicine Technology to Provide Remote Hospice/Palliative Care Services To convey the process of providing **hospice/palliative** medicine services **remotely** using clinical video telemedicine technology”. . . “in various treatment sites (**remote** clinics, home visits) and with various diseases (cancer, COPD, CHR, ALS, **dementia**)”	Palliative care teams at a few VA Medical Centers throughout the country	Rural-Urban •R ural definition not provided but states: “services can be provided at great distances from large urban medical centers. This can be particularly helpful in the **rural** setting”	• Authors identify that these services can be effectively used for various diseases such as cancer, COPD, CHF, ALS, dementia) in rural areas where “driving hundreds of miles to provide a patient with palliative care services is not feasible, practical or cost effective” • Services are helpful and effective within short distances by reducing “the burden of patients traveling to local medical centers when illness progression limits the ability to even leave the home without great burden)
Haverhals et al. [29] 2018 United States	Conference abstract	Connecting care way out there: using video visits to provide VA specialty care for veterans at state veterans homes in Colorado and Oklahoma To demonstrate the feasibility of increasing access to care for **rural** veterans living in state-run veteran nursing homes (SVHs) via video visits to veteran affairs medical centers (VAs)	Providers from 3 SVHs in rural Colorado and 7 in rural Oklahoma were trained in telehealth visits in geriatrics, **palliative care**, mental health, wheelchair, psychiatry and **dementia**; 3 nurse care coordinators were involved in pre- post-visits (video visits) with veteran n = 15 visits in Colorado and n = 63 in Oklahoma qualitative interviews with veterans, providers, and staff, field notes, cost savings analysis	Rural	• 100% of n = 15 video visits in rural Colorado were technologically successful, with 18 referrals made to other specialists during these visits • 19 of the n = 63 video visits in rural Oklahoma were psychiatry visits (n = 47 were ‘wheelchair clinic visits’) • “costs analyses indicate an estimated savings of $68.64 per video visit substituted for an in-person visit at rural SVHs” • “cultivating buy-in from key stakeholders at SVHs and VAs in geriatrics, telehealth, administration, social work, and palliative care, has been key to success” along with regular meetings and communication with all those involved • Anticipation of issues around scheduling visits and administrative processes is key to setting timelines/goals • Provision of technical support to VA providers delivering care via video visits is key to success and sustainability of intervention
Hall et al. [30] 2016 Scotland	Conference abstract	Technology Enabled Care (TEC) provision for the care home sector in the Scottish Highlands: video conferencing in care homes **Technology-Enabled Care (TEC) via nurse-led psychiatric units are introduced to 3 rural care homes via video-conferencing** to provide improved access to psychiatric care services, to address challenges of access to specialist psychiatric expertise for staff and for residents affected by **dementia** in rural areas due to long gaps between consultant visits and the anxiety and stress related to travel to secondary care for appointments in between consultant visits.	Stakeholder groups involved in the psychiatric clinics included the service provider (NHS Highland), technology enabler and facilitator (Scottish Centre for Telehealth and Telecare, SCTT), care home managers and staff, care home residents/family members, occasional care home users (day care and lunch clubs) and other wider community users such as GPs, social workers, hospital consultants, pharmacists, community nurses	Rural	• Impact on residents: quicker assessment, treatment review and regular monitoring; residents and family members reported this approach is “more responsive to their needs” • Impact of specialist access on care home staff: increased knowledge, experience and advice increased confidence and skills, more active involvement in care, and more “valued in their role” • Care homes have become more able to manage “complex cases and challenging behaviours at the local level resulting in less likelihood of patients being admitted to hospital and remain cared for in the care home; when hospital admission was necessary, earlier and more frequent follow-up often resulted in residents being able to be discharged back to the care home • More frequent follow-ups also allowed for faster medication adjustments, with some residents becoming managed via behavioural plans • Facilitators of successful implementation: early stage involvement by care home staff; training for as many potential users as possible allowed for technology to be applied in other areas beyond the psychiatric clinics • Authors concluded that: similar input and support for residents without using this approach would involve significant travel time and costs for residents/families to access specialist services; for care home staff, accessing similar amounts of relevant training would not be possible due to limited resources • Residents and families benefitted from both the individual consults as well as the knowledge and confidence gained by care home staff; these benefits have resulted in “improved patient outcomes by early intervention through participatory care planning” • Wider community impacts are beginning to emerge where other initiatives are being launched that use the TEC such as polypharmacy review clinics, hospice palliative care and the way the local community are using the equipment to access a range of specialist services
Hall, K. [31] 2016 United States	Conference abstract	To present a palliative care vision for people with Parkinson Disease (PD), created by people with PD and their families as stakeholders as participants in a palliative care research study	Key items presented: “confusion about the palliative care term, benefits of a unified, consistent approach to palliative care for people with PD, need for changes to reduce angst among people with PD and their caregivers at and after diagnosis (such as with education) and better ways to reach more patients/caregivers, advance planning assistance before advanced stage, caregiver needs, under-utilization of hospice, and patient control over the circumstances of their pending death”. Also, fundamental changes in physician attitudes and training: “ethical aspects of referring patients that better meet patient/caregiver needs, taking the time to understand patient wants/needs and with sensitivity/empathy, role changes when prolonging life is no longer an option,how to address caregiver needs, consideration of alternative methods of care (such as telemedicine) for patients/caregivers in **remote** locations, recognition of short-term priorities”	Rural-Urban	• Not reported
Subramanian, I. [32] 2016 United States	Conference abstract	Approaches to providing palliative care to the PD [Parkinson Disease] community To present and discuss aspects of and approaches to providing palliative care to the PD community	Key items presented: barriers to providing **palliative** care and ways to address, describe multidisciplinary team approach to palliative care delivery in various settings, using technology to deliver palliative care to remote settings, complimentary alternative approaches to **palliative** care delivery, issues in caring for PD patients in palliative/ hospice settings and what providers need to know about PD symptoms including dementia, and how to deliver caregiver care	Rural-Urban	• Not reported
Corbett, et al. [33] 2015 United States	Conference abstract	Pilot of a teleGeriatrics Interprofessional Curriculum in Long-Term Care To be aware of and able to discuss the barriers that exist to providing optimal geriatric palliative care within rural long-term care (LTC) settings and to appreciate a new modality (teleGeriatrics) of education within LTC and explore how this could be incorporated into practice with specific areas of need regarding “geriatrics and **palliative** medicine content, especially **dementia** and delirium and interprofessional team training” to develop competence in these areas To pilot a video-teleconference “teleGeriatrics” curriculum for rural LTC teams and to compare learners’ response to video-teleconference versus in-person team training by assessing interprofessional team training impact on 1) attitudes toward teams, 2) self-perceived team-work skills, and 3) knowledge of and comfort with **dementia** and delirium	Quasi-experimental design: Teams randomized to each group Convenience sample of VA staff from LTC including hospice and palliative care, skilled nursing care, **dementia** care, and post-acute rehabilitation. Learner and course evaluations (comparisons both within and between groups)	Rural	• Positive attitudes increased significantly by 15.9% (p < 0.0001) • Positive perception of team efficiency increased significantly by 15.9% (p < 0.0001) • Participants confidence in own team-based care skills significantly increased by 16.7% (p < 0.0001) • Geriatrics and palliative medicine knowledge on post-test improved across all areas (p < 0.05) • Most participants reported teleGeriatrics was as effective as in-person training (78%) but still showed preference for in-person training (41%)
Herman, et al. [34] 2013 United States	Conference abstract	Improving rural access to dementia and consultative palliative care through telehealth: project ECHO The goal of project ECHO (Extension for Community Healthcare Outcomes) is to train physicians to safely and effectively manage geriatric-focused diseases in **rural** areas via telehealth	45 clinics held with 553 attendees from 8 health professions. Pre-post surveys Focus groups ECHO achieves its goal by: 1) information technology to support scarce healthcare resources; 2) “best practice” disease management model to improve outcomes; 3) case-based learning and patient co-management with university-based specialists; and 4) a centralized database to measure outcomes. Interdisciplinary teams for **dementia** and **palliative** care each provide bimonthly clinics with their community-based partners	Rural	• Acceptance of this dementia-palliative care model has been excellent • Increase in knowledge about treatment of pain as the highest rated motivator for participation • Significant improvement in self-efficacy ratings pre-post (4.34 to 5.26, p = 0.007) on 1–7 Likert scale where notable improvements included: ability to treat pain, education provision to fellow professionals about end-of-life care and management of non-pain symptoms • Focus groups for dementia care providers also provided feedback on positive benefits of participation • Author conclusions: this telehealth consultation model permits primary care practitioners to retain responsibility for patient management while assuring increasing independence and sharpening of clinical skills which leads to increased self-sufficiency in management of complex older patients; sustaining this program is contingent on “demonstrable clinical outcomes” and financial viability
Kind et al. [35] 2012 United States	Conference abstract	The VA Coordinated-Transitional Care (C-TraC) Program: A Registered Nurse Telephone-Based Initiative to Improve Transitions for Hospitalized Veterans with Dementia and Other High-Risk Conditions To test the feasibility of C-TraC, designed to improve care coordination and outcomes in hospitalized veterans with **dementia** and other high-risk conditions	n = 116 Hospitalized veterans with cognitive impairment/delirium/or dementia, or lived alone, or had prior hospitalizations, discharged to the community and were over age 65 years	Not specified	• No patients refused enrolment • More than half (52%) had medication discrepancies detected and rectified in first 48–72 hours by CTraC (average of 2 errors per patient, ranging from 0 to 9 errors per patient) • Compared to ‘usual care’, significantly more patients upon hospital discharge left with follow-up care appointments arranged (85% versus 60%, p < .001) • Compared to ‘usual care’, non-significant trends toward higher visiting nurse rates (27% versus 21%), palliative care (13% versus 8%), and hospice referral (7% versus 3%) • Authors conclusions: C-TraC is a feasible low-resource, telephone-based intervention for veterans with cognitive impairment or other high-risk conditions and merits additional outcome testing to determine its viability and effectiveness in rural or other settings challenged by distance, low-resources and patient vulnerability
Menon et al. [36] 2011 United States	Conference abstract	Telemedicine as a medical intensive care unit/palliative care tool to improve rural health care To conduct a pilot study to determine the feasibility of using telemedicine conferences with rural families of patients transferring to our medical intensive care unit (MICU) with a high risk of imminent death, to provide palliative care consultation and improve communication	Over 11 months, 12 unscripted telemedicine consultations were provided Inclusion criteria included a variety of terminal diseases (such as organ failure, advanced dementia, end-metastatic cancer, etc.) expected death to occur in less than 6 months Outcomes measured included patient death, transfer rates, and ultimate disposition. Physician attitudes and satisfaction with intervention	Rural	• 10 patients (83%) died and 8 patients (67%) transferred to tertiary care facility from their respective hospitals • 7 patients (58%) returned to their hometown after a short stay in tertiary care technical limitations and issues to evaluate in future were identified including **remote** coverage to conduct teleconferences and technical assistance at rural hospital level • Authors’ initial conclusions: palliative care consultation can be effectively carried out by telemedicine for critically ill patients however, preparation and technical expertise are key to successful implementation; more patient/family satisfaction data are needed
Global Health Observatory Data Repository, Global Dementia Observatory (GDO), World Health Organization (WHO) [37] 2017 International	Indicator Metadata Registry	Accessibility of **palliative and end-of-life care** services in community for **dementia** [Capital/capital and main cities/capital, main cities, **rural** areas]	**Data repository of health-related statistics for 194 member states** **IMRID:** 5108 **Dementia Indicator ID:** 8.6.1 **Original Submeasure:** What is the accessibility of these services? **Data Type Representation:** Categorical **Rationale:** “The needs and preferences of people with dementia can be met and their autonomy from diagnosis to the end of life respected through integrated, culturally-appropriate, person-centred, community-based health, psycho-social, long-term care and support services and, where appropriate, the inputs of families and carers.” **Definition:** “Identifies the accessibility of palliative care services for dementia in different locations including: capital only, main cities/capital only, main cities and rural areas. Palliative care is an approach that improves the quality of life of patients and their families facing the problem associated with life-threatening illness, through the prevention and relief of suffering by means of early identification and impeccable assessment and treatment of pain and other problems, physical, psychosocial and spiritual.” **Method of estimation:** “Inventory of currently implemented and available services and supports for people with dementia provided by the national authority's response”	Rural	• Of the 21 reporting participating member states, as of November 28, 2017, one-third (n = 7 countries) identified as having available palliative and end-of-life care services for dementia in the community in rural areas (Australia, Chile, France, Italy, Netherlands, Sweden, and Switzerland) • Note: no other rural-specific data (including no rural-specific data for hospice centers *[both dementia-specific and non-specific])*
Dementia.org.au [38] 2018 Australia	Media Release	South Australian election candidates called on [by **Dementia** Australia] to commit to increased regional support and improved training in aged care	N/A	Rural-Urban	• Dementia Australia (DA) called on the political sector for policy and funding commitments prior to election to tackle dementia 5 Calls Included: • 1) $700,000 for **rural and regional dementia support via Dementia Link Workers** • 2) $1 million **for creation of Dementia-Friendly Communities** in three main areas of South Australia • 3) $550,000 to improve End of Life Care via Nightingale Nurses • 4) $250,000 for more training/support for South Australian workers in aged care to increase understanding of dementia • 5) Commitment to call for a national funded dementia action plan • Many people with dementia end up in hospital receiving unwanted interventions instead of needed palliative care wanted either at home or in care home; access even more limited for rural/remote and other unique characteristics (social/cultural/language) • Dementia Australia’s Nightingale Nurses in South Australia help fill these key gaps and more funding will help with adding 4 more **“specialized dementia nurses to support the unique palliative care needs of south Australians living with dementia”** • Dementia-friendly communities help people with dementia stay at home where most want to be and for longer, decreasing costs to health/aged care system and deservedly increasing their quality of life; South Australia wants to make it a “dementia-friendly society”
Johnston, J. [39] United States [date unknown]	Personal Reflection	Being Patient: The latest news on **Alzheimer’s disease** (AD) and brain health research–The Memories Project Personal reflection on caring for her **rural**ly-retired father who had **Alzheimer’s** and mother who had cancer	Daughter’s reflection of parent’s dementia journey	Rural-Urban	• **Long-distance caregiver for father with AD** and in-person caregiver for mother with cancer, each of whom died in 2011 • Had only **limited knowledge of AD** and cancer and felt “overwhelmed and unprepared” • Experience led her to become an advocate for family caregivers who are “often invisible and neglected by our government and society”. . . “often aren’t heard”. . . and “difficult for them to find help if they live in a rural area with few resources or if they feel isolated” • Created “Memories Project” to deal with grief after father with AD died, to connect with others going through similar things and advocate for AD awareness • Memories Project involved blogging one memory of him each day for one year • Aging and managing illness/disease in rural areas comes with “own challenges related to finding care”; often non-existent or limited services taken for granted in urban settings • Father was placed at closest memory care center available 1.5 hours away; spousal caregiver had to commute there by bus • Near end-of-life, local hospital “could not meet his complex medical needs” and so he was transferred more than 3 hours away into intensive care unit so spouse could not travel as often as wanted in final weeks of life • Caregiving in rural areas requires building a support network for people with dementia which can help with issues (eg., wandering, respite care); “establishing connections” with neighbors, etc. helps people in community to watch out for them • **Technology in rural setting** can help too (eg., reducing safety risk with fall protection device)

**Table 4:** Peer-reviewed and grey literature mapped to themes.

Themes Studies	Knowledge about dementia	Availability, accessibility, and utilization of palliative and end-of-life care services and supports	Decision-making about care, the value of a person-centered approach and collaborative support	Perspectives on artificial nutrition, hydration, and comfort care	Quality of life and death
**Peer-reviewed n = 12**					
**De Vleminck et al. 2018 [16]**		**✓**			
**Lethin et al. 2018 [17]**	**✓**	**✓**			
**Volandes, et al. 2011 [18]**	**✓**		**✓**	**✓**	
**Miller et al. 2010 [19]**		**✓**			
**Mitchell et al. 2007 [20]**		**✓**			
**Gessert, Haller et al. 2006 [21]**		**✓**	**✓**	**✓**	
**Gessert & Calkins 2001 [22]**			**✓**	**✓**	
**Smith et al. 2016 [23]**	**✓**		**✓**	**✓**	**✓**
**Forbes et al. 2012 [24]**	**✓**	**✓**	**✓**		
**Lindsay et al. 2010 [25]**	**✓**	**✓**	**✓**		**✓**
**Modi et al. 2010 [26]**	**✓**		**✓**	**✓**	**✓**
**Gessert, Elliott, et al. 2006 [27]**			**✓**	**✓**	**✓**
**Grey n = 12**					
**Franklin et al. 2018 [28]**		**✓**			
**Haverhals et al. 2018 [29]**		**✓**			
**Hall et al. 2016 [30]**	**✓**	**✓**	**✓**		
**Hall, K. 2010 [31]**	**✓**	**✓**	**✓**		
**Subramanian et al. 2016 [32]**	**✓**	**✓**	**✓**		
**Corbett et al. 2015 [33]**	**✓**	**✓**			
**Herman et al. 2013 [34]**	**✓**	**✓**	**✓**		
**Kind et al. 2011 [35]**		**✓**			
**Menon et al. 2011 [36]**	**✓**	**✓**			
**World Health Organization 2017 [37]**		**✓**			
**Dementia Australia 2018 [38]**	**✓**	**✓**	**✓**		**✓**
**Johnston, J. (nd) [39]**	**✓**	**✓**	**✓**		**✓**

Most peer-reviewed studies were quantitative (*n* = 7) [16–22], with publication dates ranging from 2001 to 2018, six of which focused on both rural and urban populations [16,17,19–22]. Quantitative study designs included three retrospective cohort [16,20,21], two retrospective cross-sectional [17,22], one retrospective longitudinal and cross-sectional [19], and one randomized control trial [18]. Five peer-reviewed studies were qualitative [23–27] published from 2006 to 2018, four of which focused solely on rural populations [23–26]. Qualitative study designs employed the following methods: focus groups [23,26,27], in-person interviews [24], and a biographical narrative [25]. Studies were most commonly conducted in the United States (*n* = 9) [16,18–23,26,27]. One was conducted in each of Australia [25], Canada [24], and Sweden [17]. Overall, studies involved more female participants than males [16,18–23,25–27]. One study had more males than females [24] and in one study participant sex or gender was not reported [17].

Grey literature included mainly conference abstracts (*n* = 9) [28–36], and one of each of the following: metadata table [37], media release [38], and personal reflection [39]. Conference abstracts were published from 2011 to 2018 and were primarily from the United States (8/9) [28,29,31–36]. Five of these had a focus on rural populations [29,30,33,34,36], three on rural-urban [28,31,32], and one [35] that was unspecified. One abstract [30] was from Scotland which had a rural focus. The 2017 metadata table contained global rural data from the Global Dementia Observatory, World Health Organization Indicator Metadata Registry [37]. The media release [38] was from Australia dated March 14, 2018 and the personal reflection [39] was from the United States (no date). Both of these involved a rural-urban focus.

Sex and gender was not addressed in the grey literature; however, almost all of the peer-reviewed studies reported on the sex of participants and none reported on gender. Overall, there were greater numbers of female participants in all but one study [24], and in one study sex was not reported [17]. Sex differences in outcomes were identified in four studies [18–20,22]. Specifically, hospice recipients with dementia were generally female [20], as were hospice decedents with advanced dementia [19]. Among nursing home residents, being male was significantly associated with feeding tube use [22] with females over three times more likely to receive comfort care versus feeding tubes [18].

The following themes emerged: 1) Knowledge about dementia, 2) Availability, accessibility, and utilization of palliative and end-of-life care services and supports, 3) Decision-making about care, the value of a person-centered approach and collaborative support, 4) Perspectives on artificial nutrition, hydration, and comfort care, and 5) Quality of life and death. Each of the peer-reviewed studies and grey literature are mapped to themes (Table 4) and their frequency of occurrence by theme is illustrated graphically (Fig 3).

### Knowledge about dementia

#### Peer-reviewed studies

Six peer-reviewed studies reported on knowledge about dementia which varied among people in general living in the community, people with dementia and their families, and health care and service providers [17,18,23–26]. Three of these studies explored knowledge about life-prolonging care [18,23,26].

A Swedish study that examined dementia care and service systems in nine municipalities reported on the educational level of professional care providers such as physicians, specialists, nurses, occupational therapists, physiotherapists, and social workers [17]. Professional care providers with specialized knowledge about dementia were reportedly rare, and those with such knowledge were typically employed in residential homes [17]. Further, the educational level (Master’s, Bachelor’s, short-cycle tertiary, post-secondary non-tertiary, upper secondary) of professional care providers was lower in general for those working in palliative care compared to those involved with screening, diagnostics, and treatment [17]. Home healthcare nurses reported feelings of helplessness in their [lack of] knowledge about how to help people with dementia [23].

Healthcare providers acknowledged the importance of informing patients and family caregivers about end-of-life care to facilitate discussion about their expectations and discuss available treatment and support options [24]. Professional care providers stated that although caregivers were often grateful for having these discussions, many avoided them and instead accessed information through avenues such as community organizations, family members involved in health care, support groups, and the internet [24]. In a case study of one rural couple living with dementia, information was provided by a multidisciplinary community dementia care service through regular discussion with a case manager to identify and address their changing needs over the course of one year prior to the death of the spouse with dementia [25].

Among older rural adults, goals of care (life prolonging, limited, or comfort care) in advanced dementia were associated with levels of health literacy, where those with the highest health literacy levels were more likely (12x) to choose comfort care compared to those with the lowest health literacy levels [18]. Knowledge about feeding tubes was found to be similarly high (average 91%) between African Americans and Caucasians [26].

#### Grey literature

Eight grey sources included information about dementia knowledge [30–34,36,38,39]. Five of these were conference abstracts that presented technology-assisted remote delivery of information and education, that could be optimally used in rural or remote settings [30,32–34,36]. These focused on providing information and education to health care providers in primary care [34] and long-term care settings [30–33], and to patients and families in hospital settings [36].

Positive effects of learning among health care providers included improvements in skills [30,33,34], perceptions of team-based care [30–33], attitudes [33], perceptions of self-efficacy and confidence [33,34], feeling more valued in their role [30], and improvements in knowledge related to geriatrics and palliative care [33,34], including their ability to share this knowledge with other providers [34]. Rural care home staff and residents acknowledged that the ability to access dementia-related specialist expertise remotely allowed for faster assessment and treatment management [30]. Various technology-assisted methods were discussed as a means to effectively address dementia knowledge and information needs in rural and remote areas [30–32] and enhance the ability of healthcare providers to provide better care [32]. Among health care providers who received remote education, more than half reported this modality was as effective as in-person training [33]. Key knowledge-related issues were presented regarding palliative care specifically intended to improve quality of life for people living with Parkinson’s Disease (PD) and its range of associated symptoms [such as PD dementia] including confusion about the term ‘palliative care’, a lack of patient and family education post-diagnosis, and physician attitudes and training [31].

The importance of increasing knowledge and education about dementia care for aged care providers and family members was also highlighted in a 2018 media release and personal reflection written by a daughter caregiver, respectively [38,39].

### Availability, accessibility, and utilization of palliative and end-of-life care services and supports

#### Peer reviewed studies

Seven studies reported findings related to dementia-related palliative and end-of-life care, support services, and/or preferred care settings [16,17,19–21,24,25]. Three of these studies were focused on hospice care [16,19,20].

Across rural and urban participants, availability and utilization of care services were lower for palliative care compared to screening, diagnostics, and treatment [17]. Although palliative care was largely available and well-utilized in most of the municipalities under study within the home, in nursing homes, residential homes, and institutional care, dementia-specific care was less available [17]. Where dementia-specific care units were available, they were used by most study participants [17]. Advance directive services were available in just two of the nine municipalities involved and were available across all disease stages however, whether these municipalities were rural or urban was not identified, and few people reportedly used these services and only did so during the end-of-life stage [17]. Rural nursing homes were smaller and less likely ‘for profit’ compared to urban nursing homes and rural nursing home residents with severe dementia were more likely than were urban residents to have advance directives [21]. Overall greater service use was associated with being nonwhite and having had a stroke [21]. Having an advance directive in place was associated with less likelihood of hospitalization and intensive care for both rural and urban nursing home residents [21].

Hospices caring for people with dementia were more likely for-profit, larger, provided care for five or more years, and served more patients from nursing homes [16]. Among discharged hospice patients, patients with dementia were more likely to have long stays compared to patients without dementia, particularly in smaller hospices [16]. Hospice access and use among nursing home decedents with advanced dementia tripled between 1999 and 2006 and length-of-stay increased from 46 to 118 days however, the lowest rates of hospice use for this group were found among states described as ‘more rural’ [19]. Compared to the mild-moderate dementia group, advanced dementia hospice decedents were more often younger and female, with advance planning in place regarding do-not-hospitalize or do-not-resuscitate orders, and had stays of 90 days or more [19]. Findings from the Family Evaluation of Hospice Care survey showed that just 12% of hospices were identified as rural and hospice recipients with dementia were more likely female, had a longer length of stay, and were less likely to receive in-hospital or for-profit hospice care, compared to those with other diseases [20].

Information about access to end-of-life care services and supports for rural people living with dementia often came from other unspecified family members, health centers, physicians, community care organizations, support groups, and the internet [24]. A case study described access to and utilization of a local multi-disciplinary community dementia care service that effectively met the needs of a rural couple living with dementia whose preferred care setting was to remain in their home throughout the end-of-life stage [25]. The non-institutional care options provided (such as ongoing support via telephone and in-home) allowed the wife to provide end-of-life care to her husband at home until his death [25].

#### Grey literature

All of the grey literature referred to availability, accessibility, and utilization of relevant care and support services, and/or preferred care settings [28–39]. Most of these (9/12) were conference abstracts that referred to the potential for [31,32], or implementation of [28–30,33–36] technology-assisted care support or service delivery for people with dementia and their families, particularly for those living in rural or remote areas.

The need for increased access to palliative or end-of-life care services and support was reflected in a 2018 media release from Australia that referred to a political call for increased funding of the Nightingale Nurses program to improve end-of-life care and support dementia care and support in South Australia [38]. Indicator metadata [37] from the World Health Organization identified just seven countries as having available palliative and end-of-life care services for dementia in the community in rural areas (Australia, Chile, France, Italy, Netherlands, Sweden, and Switzerland). In the United States, a daughter’s personal reflection of her rural parents’ dementia care journey described the limited options for local care and the effect this had on the ability of family to visit, and identified a need for support networks in rural areas [39].

Remote technology was presented as a potential means to increase access to palliative or end-of-life care services or supports for people living with dementia [28–36], especially those in rural areas, which might allow them to remain in their preferred care setting (i.e. place of residence or in their local community). Telehealth and other videoconferencing methods to deliver specialist care in rural areas often results in benefits to both healthcare providers and people with dementia and their families, including more frequent follow-up, and increased capacity of providers to manage challenging cases locally which may reduce hospitalization [30] and patient and family travel burden [28,30,35]. Strategies for successful sustainability of remote specialist care delivery include buy-in from key stakeholders, anticipation of potential scheduling and administrative issues, ongoing communication, and provision of tech support [29]. Participation in a telephone-based initiative for community-living veterans with dementia and other high-risk conditions upon discharge from hospital increased palliative care and hospice referrals by 5% and 4%, respectively [35].

### Decision-making about care, the value of a person-centered approach and collaborative support

#### Peer-reviewed studies

Eight studies reported findings related to palliative or end-of-life care decision-making, a person-centered approach and/or collaborative support for people with dementia [18,21–27]. Four of these were focused on preferences for life-sustaining options [18,22,23,26].

Decision-making for persons with dementia and their care partners typically involved consultation with their physician and other family members [24]. Decisions were at times delayed by family members who avoided conversations about the future [24]. The importance of person-centered, collaborative care in decision-making was illustrated in a rural case study where a couple living with dementia decided to discontinue residential respite care [25]. Support was provided in-home using a person-centered, case-management approach within a community collaborative model of care for end-stage dementia. Collaboration between the couple, their case manager, and other care providers, along with a focus on the desires and needs of the couple, facilitated spiritual reflection and a sense of relief during end of life at their home.

Both rural and urban family members of older adults with dementia living in a nursing home identified as advocates for their family member, and expressed few desires regarding death beyond hoping it would come both quickly and peacefully [27]. A study that examined end-of-life service use for those with severe dementia in rural and urban nursing homes reported that during the last 90 days of life, rural nursing home residents were more likely than urban residents to have do-not-resuscitate orders and/or living wills and less likely to have used feeding tubes [21].

Decision-making about end-of-life care was reportedly influenced by a decision-aid video among rural older adults who participated in a randomized control study [18]. Those who viewed the decision-aid video (the intervention group) were more likely to opt for comfort care (symptom relief) (91%) compared to the control group (70%) [18]. Nine percent of the intervention group opted for limited care (hospitalization but no life-saving measures) versus 5% of the control group and none of the intervention group opted for life-prolonging care compared to 16% of the control group [18].

An exploration of views about end-of-life care among rural community members showed that while they wanted their desires and beliefs considered in end-of-life decision-making, adequate information to help make such decisions was lacking [26]. While many participants recognized the value of early planning and decision-making, there was concern about whether advance directives would be implemented or overruled by health professionals [26].

#### Grey literature

Six grey sources referred to decision-making, the importance of a person-centered approach, and/or collaborative support [30–32,34,38,39].

Introducing video conferencing technology in rural care homes to provide specialist psychiatric consultation for staff and residents allowed for quicker assessments, treatment review, and regular monitoring which residents and family members recognized as being “more responsive to their needs” [30]. Regular collaboration with specialists using a telehealth consultative model for dementia and palliative care in rural areas was reportedly beneficial, and authors suggested this increased the capacity of primary care practitioners to retain responsibility for patient management [34]. Finding alternative ways (e.g. via technology) to reach patients and caregivers is also necessary to facilitate exploration of their wants and needs and advance planning before later disease stages [31] and should include a multidisciplinary team approach to delivery of palliative care in remote areas [32].

In South Australia, another approach to improve palliative care for people living with dementia in rural areas is specialized support provided by “Nightingale Nurses” [38]. This approach allows for persons with dementia to remain cared for in-home when this is their preferred care setting [38]. Rural settings were referred to as often ill equipped to deliver end-of-life care and meet the complex, individual needs of people with dementia, and building a collaborative support network was seen as essential [39].

### Perspectives on artificial nutrition, hydration, and comfort care

#### Peer-reviewed studies

Six studies reported on perspectives about artificial nutrition, hydration, and/or comfort care during palliative or end-of-life stages of dementia [18,21–23,26,27].

Two studies reported differences in feeding tube use among rural and urban nursing home residents with dementia in the United States, both of which found increased use of feeding tubes among urban residents [21,22]. First, using data for nursing home residents over age 65 years with severe dementia living in rural, midsize, and urban counties in Kansas between January 1, 1994 and June 30, 1998, feeding tube use was greater among urban nursing home residents (19.3%) compared to nursing home residents in midsize (8%) and rural (6.4%) counties [22]. Specifically, the use of feeding tubes was associated with living in an urban nursing home, being nonwhite (compared to white) and male (versus female), having either Alzheimer’s disease or stroke and a greater level of dependency as well as having difficulties with swallowing [22]. Second, rural residents were less likely than urban residents to be hospitalized for long periods or in intensive care and less likely to use a feeding tube in the last 90 days of life [21]. Further, use of feeding tubes and services in general were most common among nonwhite urban nursing home residents [21].

Home healthcare nurses working in rural North Carolina were interviewed regarding their perspectives of artificial nutrition and hydration for people with late stage dementia [23]. Some nurses identified this as a complex issue in end-of-life decision-making with difficulty in determining the degree of the patients’ pain and suffering when they have lost their capability for ‘purposive language’ [23]. Most nurses expressed a focus on providing comfort for both patient and family where artificial nutrition and hydration were used as comfort measures for both. However, many nurses admitted feeling that while these measures can extend a patient’s life and provide comfort to the family, they can also increase patient suffering (i.e. painful procedure, risk of patient restraint to avoid tube removal, risk of infection, aspiration, discomfort, nausea, vomiting, and diarrhea) [23]. Many nurses acknowledged that although they felt such measures were futile, they always discussed options and considered the wishes of patients and their families [23].

Among interviewed families of rural and urban nursing home residents with dementia, most rural participants were against the use of life-sustaining measures simply to delay death whereas many urban participants were open to such measures and preferred more ‘aggressive’ treatment [27]. African-American and Caucasian community members living in ‘largely rural’ areas expressed similar concern and support in general regarding feeding tube use for people with dementia during end-of-life [26]. While many perceived tube feeding as prolonging suffering, others noted its importance in preventing starvation, providing comfortability, and extending life until all family members could be present [26]. Some also noted the important symbolism of food and caring where denying food to a sick person goes against human nature [26]. Others noted that decision-making about feeding tube use can be impacted by providing information about goals of care [18] and should involve consideration of key individual factors such as age and cognitive status [26].

#### Grey literature

No articles in the grey literature reported on perspectives about artificial nutrition, hydration, and/or comfort care during palliative or end-of-life stages of dementia.

### Quality of life and death

#### Peer-reviewed studies

Four studies, all qualitative, discussed the quality of life and death during palliative or end-of-life stages of dementia [23,25–27].

Rural home health nurses believed that quality of life can be negatively impacted by prolonging patients’ suffering through the use of life-sustaining measures such as artificial nutrition and hydration [23]. Rural African-American and Caucasian community members acknowledged that when known, honoring the wishes of the person with dementia was important and many reported valuing a natural death without life-sustaining measures where tube feeding was described as worse than death [26]. Many also expressed religious views of life and death as being in God’s hands [26].

Many rural and urban family members of residents with dementia living in nursing homes were more accepting of eventual death versus imminent death [27]. Most rural family members described an unconditional acceptance of death as a natural process they hoped would be both quick and peaceful [27]. While urban family members (many of whom did not refer to ‘death’ at all) also wished for a peaceful death, they also placed conditions on their acceptance of death, particularly in terms of the timing [27]. As illustrated in the case study of a person centered, community collaborate model of end of life care, such supports have the potential to enhance spiritual reflection and comfort during end of life [25].

#### Grey literature

Two grey sources reported content related to the quality of life and death during palliative or end-of-life stages of dementia [38,39].

In a 2018 news release, Dementia Australia called for policy and funding to support services such as specialized dementia nurses for rural palliative and end-of-life care, to help people stay in their preferred care setting thereby increasing their quality of life [38]. Similarly, a personal reflection mentioned the importance of addressing the care needs of people living with dementia in rural settings, to avoid non-local placement which decreases the quality of end-of-life for the person with dementia [39].

## Discussion

Despite the growing numbers of people living with dementia around the world, the need for knowledge about dementia in general and as a terminal disease with an unpredictable trajectory remains. In this review, we identified three key areas for improvement, including: increasing knowledge about dementia in general, especially about the advanced and end-of-life stages of the disease, among health care providers, people with dementia, their families, and caregivers; having informed, early conversations and decision-making about palliative and end-of-life care options; providing a person-centered approach and allowing for palliative and end-of-life care to occur in preferred care settings. These key areas are discussed in terms of access and use in rural areas, and the potential for technological solutions to address these issues. Other findings of this review are considered, including rural-urban differences, the impact of sex and gender, and gaps in the literature.

### Access and use of palliative and end-of-life care supports

The lack of available, accessible health services in general in rural settings due to geographical barriers is well-known [40]. The World Health Organization (2017) reported just seven countries as having palliative and end-of-life care services for dementia available at the community-level in rural areas [37]. In this review, the availability and utilization of palliative and end-of-life care services for people with dementia and their families was reported as lower compared to that of screening, diagnostics, and treatment [17]. Further, although hospice access, use, and length of stay among nursing home decedents with advanced dementia was reportedly increasing, rural areas had the lowest rates of use [19], rural hospice care was reportedly lacking in general [20], and service use appeared to be relatively mixed. In addition, this review identified the need to overcome rural barriers to providing person-centred, collaborative care, to enable provision of palliative and end-of-life care in-home [25,38,39]. Such options are feasible by increasing home visits and utilizing modern technology, thus increasing equity in care provision [41]. Alternative methods of delivering care and support were presented within the literature synthesized in this review, such as providing in-home visits and telephone support [25] and using remote technology [28–36]. Using alternative methods to increase support and service access was perceived to facilitate the ability of people with advanced dementia to remain cared for in their preferred care setting and community [28–36].

### Potential for technological solutions in rural areas

Alternate ways to deliver health care services and supports that are not face-to-face continue to be developed and implemented across both rural and urban areas to improve accessibility, availability, and utilization [42] and more specifically, for rural people living with dementia [43–46]. Key benefits of using remote technology in this review included increased provider capacity to gain and provide specialist expertise and retain management of patient care, more frequent follow-ups, decreased likelihood of hospitalization [30], and reduced travel burden for patients and families [28–30,35]. This review supports the findings of previous reviews regarding the desire to remain cared for at home during end-of-life [47] and the benefits of technology to support care delivery, including education, in the home, particularly in rural areas that are limited by distance, isolation, and transportation [40]. Potential barriers to using technology in this way were also identified in this review such as a lack of key stakeholder buy-in, scheduling, and tech-support [29], which are similar to barriers reported in a recent systematic review regarding technology-based interventions to support healthcare [48].

In this review, providing various methods of delivery of information, care, and support services enhanced the ability of people with dementia and their families to make collaborative, informed decisions. Overcoming barriers to early decision-making is vital and can be facilitated in various ways, including the use of technology to collaborate remotely with specialists [30,34]. A recent review of decision aids to assist family caregivers of people with advanced dementia with end-of-life decision-making found that often technology was underutilized, in spite of the advances made in information and communication technologies (such as interactive websites or mobile apps) [49].

### Sex and gender differences

Most of the peer-reviewed literature considered sex in their analysis and differences were found in four studies [18–20,22]. Firstly, hospice recipients with dementia [20] and decedents with advanced dementia [19] were more frequently female [20]. It is possible these findings could be attributed to the longer life span of females in general [50] and that the risk of dementia increases with age however, it is likely that there are more complex factors involved [51]. Secondly, among nursing home residents with advanced dementia, females were far more likely to receive comfort care (more focus on symptom control and pain relief) versus feeding tubes (more focus on prolonging life) than males [18,22]. Other studies have shown similarly high rates of feeding tube use among males compared to females but the purported reasons for this are unclear [52,53]. Both studies also reported that men were slightly younger than the women [52,53], and one of these studies found that younger age was also associated with better functional status [53].

Continuing to include sex- and gender-based information and analysis in future studies on palliative and end-of-life care for rural people living with dementia is important to identify any existing differences, which could support the development and implementation of more tailored services and supports that would effectively address needs and preferences that differ across sex and gender.

### Rural-urban differences

In studies that examined both rural and urban populations, some rural-urban differences were reported [21,22,27]. One study found that among nursing home residents with severe dementia, rural nursing home residents compared to urban were more likely to have advanced directives in place and were less likely to be hospitalized for long periods or in intensive care [21]. Similar findings were reported among all three studies where rural participants were less inclined to use feeding tubes compared to their urban counterparts. Specifically, two studies found that among nursing home residents with severe dementia, rural nursing home residents were less likely to have used feeding tubes [21,22]. The third study found that among the families of rural and urban nursing home residents with dementia, most rural family members expressed opposition to the use of life-sustaining measures to delay death whereas many urban family members were more open to such measures [27].

It is perhaps not surprising that the topic of nutrition care at end of life was found to be a concern in this review. The aim of nutrition care at end of life is to provide comfort, and artificial nutrition and hydration can be an invasive measure that increases discomfort, and for these reasons is often not recommended for dementia palliation [54–58]. Little is known about the rural-urban differences in attitude related to artificial nutrition and hydration at end of life. In this review, the attitudinal differences demonstrated between urban and rural represent a facet of end of life care that warrants further inquiry. A better understanding of the reported differences between rural and urban preference for artificial nutrition and hydration at end of life is urgently needed.

In one study, most rural family members viewed death with unconditional acceptance, as a natural process while many urban family members were less inclined to mention death at all and placed conditions on their acceptance of death, especially in terms of when death would occur [27]. These findings are also consistent with those found in two systematic reviews that suggested rural people may have a more deterministic view of illness [40] and that rural people were more accepting of ill health in general, where ‘good health’ was regarded more in terms of maintaining independence, the ability to work, and their social relationships [59]. Factors that affect attitudes toward death and dying include previous experience, culture, and social networks [60]. This could explain rural-urban differences where experiences, culture, and social connectedness may be distinct in rural communities. Although not all rural areas are inherently the same, rural communities may share more common experiences, beliefs, values, and customs, compared to their more diverse urban counterparts.

### Peer-reviewed–grey literature differences

The inclusion of grey literature is a strength of this review which allowed the authors to identify differences and similarities with that of the peer-reviewed literature (which are illustrated by theme in Table 4). Although 75% of both the peer-reviewed and grey literature were similarly conducted in the United States, just one (1/12) peer-reviewed study mentioned the use of remote methods of care for people with dementia and their families during palliative and end of life stages, such as telephone support [25], particularly for those living in rural or remote areas. In contrast, most (9/12) of the grey literature (primarily conference abstracts), reported on the potential for [31,32], or implementation of [28–30,33–36], remote methods such as technology-assisted care support or service delivery that appeared to be beneficial in increasing access to supports and services and remaining cared for in preferred care settings and communities.

Other differences between the peer-reviewed and grey literature also warrant mention. As noted earlier, perspectives on artificial nutrition, hydration, and comfort care were identified in half of the peer-reviewed literature included in this review (6/12) [18,21–23,26,27] but was not identified in any of the grey literature (0/12). In addition, the demand for policy and funding opportunities to address the needs of people with dementia and their families during palliative and end-of-life stages were exemplified in a 2018 media release (grey literature) [38]. Dementia Australia called on the South Australia political sector for a commitment to various dementia policy and funding that could help address these needs, iterating the importance of service and support access that would allow them to remain cared for in their own community, particularly for those living in rural and remote areas. Other literature included in this review did not speak to the necessity of such policy and funding however, this should not be interpreted as indicating a lack of need.

### Gaps in literature

This review found a lack of rural-specific research in general exploring palliative and end-of-life care for people living with dementia; this gap demands attention. Further, all of the literature included for synthesis in this review originated from advanced, industrialized nations (United States, Canada, Australia, Sweden, and the United Kingdom [Scotland]). In fact, most (75%) of the included literature originated from the United States, with no universal system of health care, and one of the richest countries in the world, which is very different from the broader spectrum of existing rural contexts across the globe. The availability of, and access to, health care in rural areas within low and middle income countries is quite different from that of more developed countries in terms of the health needs of the population and their ability to access and receive quality, affordable care by trained health professionals. People living in rural or remote areas encompass almost half of the world’s population [61] where there are a number of unique conditions that can be both beneficial (such as being supportive, close-knit communities) and detrimental (such as being geographically isolated) in terms of health [59]. Thus, there is an important gap in the available literature regarding palliative and end-of-life care for people living with dementia in rural areas in general, particularly from non-industrialized nations and from countries other than the United States, where healthcare systems can be quite different in other countries

Further gaps were also identified. The literature included in this review was lacking in evidence regarding the financial and social implications of the responsibilities involved for informal/family caregivers’ caring for people with dementia, particularly in rural areas where formal care options and resources are limited. The need for more studies on a national level with large samples of rural caregivers to explore the effects of rurality on caregiving has been highlighted in previous literature [62]. Another identified gap in the literature included in this review was regarding the use of collaborative, multidisciplinary dementia care teams. It is possible such teams are not widely available in rural areas, however one study in this review illustrated the benefits of using of such a model of care for end-stage dementia in their rural Australia community [25]. Further evidence of a similar successful model in previous literature exemplifies the importance of interprofessional dementia care teams as a plausible, effective model in rural North Carolina (United States), as best practice in dementia care where further research is needed [63]. Lastly, the absence of any definition of ‘rural’, or a common definition of ‘rural’, within the included literature illustrates and important gap in existing research, which contributes to the limited comparability described below.

### Study limitations

Although the search strategy was intentionally broad with no restriction on publication year, and grey literature was also included (a strength of this review in terms of providing a comprehensive, balanced view of the existing evidence and reducing the potential for publication bias), the search was limited to studies published in English language only. It is possible that some literature sources were missed, which could limit the generalizability of the findings. Generalizability is further limited due to the majority of the literature coming from the United States, with a lack of literature from other countries and healthcare systems to explore differences in trends or patterns. In addition, comparability was limited due to the heterogeneity of the literature included for synthesis, however, thematic analysis of the extracted findings was performed to effectively draw conclusions based on common elements. Further, no quality assessment of peer-reviewed literature was conducted, although this is consistent with the scoping review methodology.

## Conclusions

In summary, the literature synthesized in this review provided an understanding and overview of the experiences, needs, and shortfalls of dementia-related palliative and end-of-life care services and supports in rural areas. Key themes were identified and main areas for improvement were discussed. Although there was a paucity of research regarding rural palliative and end-of-life care for people with dementia, several areas were highlighted within the existing literature including the importance of extending further knowledge about dementia, having early conversations about advanced care and treatment options to allow for informed decision-making, and providing a person-centered approach to allow for individuals to remain cared for in their preferred care settings. The potential for using technological solutions to help address rural issues with access to services and supports was also discussed. These findings can be used to inform future research and policy and the development of services, supports, and intervention strategies to improve the lives of people living with dementia and further research is recommended.

## Supporting information

S1 Appendix Preferred reporting items for systematic reviews and meta-analyses extension for scoping reviews (PRISMA-ScR) checklist. (DOCX)

S2 Appendix Peer-reviewed literature search strategy. (DOCX)

S3 Appendix Grey literature search strategy. (DOCX)
