# Supplementary material for: Palliative and end-of-life care for people living with dementia in rural areas: A scoping review
Source: PLoS One. 2021 Jan 14;16(1):e0244976. doi: 10.1371/journal.pone.0244976 (PMC7808637; doi:10.1371/journal.pone.0244976)
Supplement: S2 Appendix — (DOCX) [file pone.0244976.s002.docx]

**S2 Appendix. Peer-reviewed literature search strategy.**

**Database: Ovid MEDLINE(R) and In-Process & Other Non-Indexed Citations 1946 to November 13, 2018**

Search Strategy:

| **#** | **Searches** | **Results** |
| --- | --- | --- |
| 1 | RURAL HEALTH/ or RURAL POPULATION/ or HOSPITALS, RURAL/ or RURAL NURSING/ or RURAL HEALTH SERVICES/ | 87103 |
| 2 | (rural* or remote* or isolat* or north* or "sparse population" or "sparse populations").mp. | 2369677 |
| 3 | 1 or 2 | 2369677 |
| 4 | dement*.mp. | 113052 |
| 5 | alzheimer*.mp. | 138709 |
| 6 | (lewy* adj2 bod*).mp. | 8744 |
| 7 | (creutzfeldt or jcd or cjd).mp. [mp=title, abstract, original title, name of substance word, subject heading word, floating sub-heading word, keyword heading word, protocol supplementary concept word, rare disease supplementary concept word, unique identifier, synonyms] | 8228 |
| 8 | (pick* adj2 disease).mp. | 3443 |
| 9 | ((semantic or vascular or fronto-temporal* or Parkinson*) adj2 dementia).mp. | 11777 |
| 10 | huntington*.mp. | 17571 |
| 11 | primary progressive aphasia?.mp. | 995 |
| 12 | exp dementia/ | 149448 |
| 13 | dementia/ or aids dementia complex/ or alzheimer disease/ or aphasia, primary progressive/ or primary progressive nonfluent aphasia/ or creutzfeldt-jakob syndrome/ or dementia, vascular/ or cadasil/ or dementia, multi-infarct/ or diffuse neurofibrillary tangles with calcification/ or frontotemporal lobar degeneration/ or frontotemporal dementia/ or "pick disease of the brain"/ or huntington disease/ or kluver-bucy syndrome/ or lewy body disease/ or prion diseases/ or taupathies/ | 152775 |
| 14 | or/4-13 | 237533 |
| 15 | Palliative Care/ or Terminal Care/ or palliat*.mp. | 103839 |
| 16 | terminal.mp. | 407931 |
| 17 | Terminally Ill/ or Attitude to Death/ or terminally ill patients.mp. or Hospice Care/ | 26662 |
| 18 | HOSPICE CARE/ or "HOSPICE AND PALLIATIVE CARE NURSING"/ or hospice*.mp. | 14886 |
| 19 | ((late* or end* or advance*) adj2 (stage* or life*)).mp. | 216363 |
| 20 | (advance* adj2 (care* or directive* or plan*)).mp. | 14886 |
| 21 | death.mp. or DEATH/ | 708241 |
| 22 | dying.mp. | 31505 |
| 23 | Suicide, Assisted/ or end of life.mp. | 24116 |
| 24 | Advance Care Planning/ or Advance Directives/ | 7310 |
| 25 | or/15-24 | 1361323 |
| 26 | 3 and 14 and 25 | 1478 |
| 27 | limit 26 to english language | 1412 |

**Database(s): Embase Classic+Embase 1947 to 2018 November 13**
Search Strategy:

| **#** | **Searches** | **Results** |
| --- | --- | --- |
| 1 | (rural* or remote* or isolat* or north* or "sparse population" or "sparse populations").mp. | 2484255 |
| 2 | rural health/ or urban rural difference/ or rural area/ or rural population/ or exp rural health care/ | 103739 |
| 3 | 1 or 2 | 2484255 |
| 4 | dement*.mp. | 185906 |
| 5 | alzheimer*.mp. | 216900 |
| 6 | (lewy* adj2 bod*).mp. | 16293 |
| 7 | (creutzfeldt or jcd or cjd).mp. | 12886 |
| 8 | (pick* adj2 disease).mp. | 6589 |
| 9 | ((semantic or vascular or fronto-temporal* or Parkinson*) adj2 dementia).mp. | 16239 |
| 10 | huntington*.mp. | 29188 |
| 11 | primary progressive aphasia?.mp. | 2224 |
| 12 | exp dementia/ | 329079 |
| 13 | dementia/ or alzheimer disease/ or cadasil/ or diffuse lewy body disease/ or diffuse neurofibrillary tangles with calcification/ or hiv associated dementia/ or huntington chorea/ or kluver bucy syndrome/ or multiinfarct dementia/ or neuronal ceroid lipofuscinosis/ or pick presenile dementia/ or presenile dementia/ or prion disease/ or senile dementia/ or tauopathy/ or progressive nonfluent aphasia/ or semantic dementia/ or primary progressive aphasia/ or Creutzfeldt Jakob disease/ or senile dementia/ or frontal variant frontotemporal dementia/ or frontotemporal dementia/ | 312980 |
| 14 | or/4-13 | 393204 |
| 15 | palliative therapy/ or palliat*.mp. | 146945 |
| 16 | palliative nursing/ | 576 |
| 17 | terminal care/ or terminal.mp. or terminal disease/ | 545303 |
| 18 | attitude to death/ or death.mp. or death education/ | 1169539 |
| 19 | dying.mp. or dying/ | 48413 |
| 20 | hospice.mp. or hospice care/ or hospice/ or hospice patient/ or hospice nursing/ | 23382 |
| 21 | terminally ill patients.mp. or terminally ill patient/ | 8952 |
| 22 | ((late* or end* or advance*) adj2 (stage* or life*)).mp. | 332121 |
| 23 | (advance* adj2 (care* or directive* or plan*)).mp. | 16943 |
| 24 | euthanasia/ or terminal care/ or end of life.mp. | 60010 |
| 25 | advance care planning.mp. or advance care planning/ | 3817 |
| 26 | or/15-25 | 2099264 |
| 27 | 3 and 14 and 26 | 2351 |
| 28 | limit 27 to english language | 2259 |

**Database(s): PsycINFO 1806 to November Week 1 2018**
Search Strategy:

| **#** | **Searches** | **Results** |
| --- | --- | --- |
| 1 | (rural* or remote* or isolat* or north* or "sparse population" or "sparse populations").mp. | 160410 |
| 2 | exp RURAL ENVIRONMENTS/ | 16261 |
| 3 | 1 or 2 | 160410 |
| 4 | dement*.mp. | 68010 |
| 5 | alzheimer*.mp. | 58430 |
| 6 | (lewy* adj2 bod*).mp. | 3612 |
| 7 | (creutzfeldt or jcd or cjd).mp. | 1094 |
| 8 | (pick* adj2 disease).mp. | 670 |
| 9 | ((semantic or vascular or fronto-temporal* or Parkinson*) adj2 dementia).mp. | 7324 |
| 10 | huntington*.mp. | 4763 |
| 11 | primary progressive aphasia?.mp. | 808 |
| 12 | exp dementia/ | 70859 |
| 13 | dementia/ or presenile dementia/ or senile dementia/ or aids dementia complex/ or dementia with lewy bodies/ or semantic dementia/ or vascular dementia/ or alzheimer's disease/ or creutzfeldt jakob syndrome/ or picks disease/ or kluver bucy syndrome/ | 70878 |
| 14 | or/4-13 | 102668 |
| 15 | exp Palliative Care/ or exp "Death and Dying"/ or exp Hospice/ or palliat*.mp. | 43562 |
| 16 | exp Terminally Ill Patients/ or terminal*.mp. | 26684 |
| 17 | hospice*.mp. | 4974 |
| 18 | exp "DEATH AND DYING"/ or exp DEATH EDUCATION/ or death.mp. or exp DEATH ATTITUDES/ | 91094 |
| 19 | dying.mp. | 34805 |
| 20 | ((late* or end* or advance*) adj2 (stage* or life*)).mp. | 36513 |
| 21 | (advance* adj2 (care* or directive* or plan*)).mp. | 4696 |
| 22 | end of life.mp. | 8813 |
| 23 | exp Advance Directives/ or advance care planning.mp. | 1886 |
| 24 | exp Euthanasia/ or exp Assisted Suicide/ | 2107 |
| 25 | or/15-24 | 153366 |
| 26 | 3 and 14 and 25 | 425 |

**Database(s): CINAHL Plus with Full Text - November 13, 2018**

Search Strategy:

| **#** | **Query** | **Results** |
| --- | --- | --- |
| S24 | S3 AND S12 AND S23  English Language | 249 |
| S23 | S13 OR S14 OR S15 OR S16 OR S17 OR S18 OR S19 OR S20 OR S21 OR S22 | 250,224 |
| S22 | ""((advance*) W2 (care* or directive* or plan*))"" | 10,897 |
| S21 | ""((late* or end* or advance*) W2 (stage* or life*))"" | 51,263 |
| S20 | (MH "Dying Care (Iowa NIC)") OR (MH "Right to Die") OR (MH "Euthanasia, Passive") OR "dying" | 18,220 |
| S19 | (MH "Death") OR "death" OR (MH "Attitude to Death") OR (MH "Death Counseling") | 141,545 |
| S18 | (MH "Advance Directives") OR "advance directive" | 5,081 |
| S17 | (MH "Advance Care Planning") OR "advance care planning" | 3,192 |
| S16 | (MH "Suicide, Assisted") OR (MH "Dignified Dying (Iowa NOC)") OR "end of life" | 18,827 |
| S15 | "terminal" OR (MH "Terminally Ill Patients") | 35,565 |
| S14 | (MH "Hospice Patients") OR (MH "Hospice and Palliative Nursing") OR "hospice*" | 17,657 |
| S13 | (MH "Palliative Care") OR (MH "Hospice and Palliative Nursing") OR (MH "Hospice Care") OR (MH "Terminal Care") OR "palliat*" | 59,370 |
| S12 | S4 OR S5 OR S6 OR S7 OR S8 OR S9 OR S10 OR S11 | 78,341 |
| S11 | (MH "Frontotemporal Dementia") OR (MH "Dementia, Vascular") OR (MH "Delirium, Dementia, Amnestic, Cognitive Disorders") OR (MH "Dementia, Multi-Infarct") OR (MH "AIDS Dementia Complex") OR (MH "Lewy Body Disease") OR (MH "Dementia, Senile") OR (MH "Dementia, Presenile") OR OR (MH "Dementia") (MH "CADASIL") (MH "Alzheimer's Disease") OR (MH "Creutzfeldt-Jakob Syndrome") OR (MH "Creutzfeldt-Jakob Disease, Variant") OR (MH "Prion Diseases") MH "Pick Disease of the Brain") OR (MH "Frontotemporal Dementia") OR (MH “HUNTINGTON’S DISEASE”) OR (MH "Frontotemporal Lobar Degeneration") OR (MH "Neuronal Ceroid-Lipofuscinoses") OR (MH “Prion Diseases”) | 7,518 |
| S10 | ""primary progressive aphasia" OR "primary progressive aphasias"" | 449 |
| S9 | "huntington*" | 2,006 |
| S8 | TI ( "pick disease" OR "pick's disease" OR "picks disease" ) OR AB ( "pick disease" OR "pick's disease" OR "picks disease" ) | 202 |
| S7 | TI ( creutzfeldt or jcd or cjd ) OR AB ( creutzfeldt or jcd or cjd ) | 967 |
| S6 | TI ( "lewy body" OR "lewy bodies" ) OR AB ( "lewy body" OR "lewy bodies" ) | 1,679 |
| S5 | "alzheimer*" | 35,165 |
| S4 | "dement*" | 52,196 |
| S3 | S1 OR S2 | 202,487 |
| S2 | (rural* or remote* or isolat* or north* or "sparse population" or "sparse populations") | 201,708 |
| S1 | (MH "Rural Health Personnel") OR (MH "Rural Health Centers") OR (MH "Hospitals, Rural") OR (MH "Rural Population") OR (MH "Rural Health Services") OR (MH "Australian Rural Nurses and Midwives") OR (MH "Rural Health Nursing") OR (MH "Rural Areas") OR (MH "Association for Australian Rural Nurses") OR (MH "Services for Australian Rural and Remote Allied Health") OR (MH "Rural Health") OR (MH "Frontier Nursing Service") | 40,581 |
