# Supplementary material for: Palliative and end-of-life care for people living with dementia in rural areas: A scoping review
Source: PLoS One. 2021 Jan 14;16(1):e0244976. doi: 10.1371/journal.pone.0244976 (PMC7808637; doi:10.1371/journal.pone.0244976)
Supplement: S3 Appendix — (DOCX) [file pone.0244976.s003.docx]

**S3 Appendix. Grey literature search strategy.**

| **Grey Database Search** | | | | |
| --- | --- | --- | --- | --- |
| **Date** | **Database name** | **Search terms**  **(located in title, abstract, keywords or combinations thereof where applicable)** | **Search results/number of items retrieved and screened for inclusion** | **Items included for synthesis** |
| Jan 29, 2019 | WHO International Clinical Trials Registry Platform | 1. Dementia OR Alzheimer 2. Palliative OR end of life OR hospice 3. Rural 4. 1) & 2) & 3) | 1) 4046  2) 1113  3) 878  4) 0 | n = 0 |
| Jan 29, 2019 | Cochrane Central Register of Controlled Trials | 1. Dementia OR Alzheimer 2. Palliative OR end of life OR hospice 3. Rural 4. 1) & 2) & 3) | 1. 14109 trials, 40 protocols, 185 reviews, 7 editorials 2. 18880 trials, 16 protocols, 497 reviews, 9 editorials 3. 7025 trials, 0 protocols, 33 reviews, 3 editorials 4. 8 trials, 0 protocols, 0 reviews, 0 editorials | n = 1 |
| Jan 29, 2019 | National Institute on Aging Clinical Trials | 1. Dementia OR Alzheimer 2. Palliative OR end of life OR hospice 3. Rural 4. 1) & 2) & 3) | 1. 187 2. 2 3. 0 4. 0 | n = 0 |
| Jan 29, 2019 | Clinical Trials.gov | 1. Dementia OR Alzheimer 2. Palliative OR end of life OR hospice 3. Rural 4. 1) & 2) & 3) | 1. 2951 2. 892 3. 522 4. 0 | n = 0 |
| Jan 29, 2019 | Open Grey | 1. Dementia OR Alzheimer 2. Palliative OR end of life OR hospice 3. Rural 4. 1) & 2) & 3) | 1. 2462 2. 1663 3. 33125 4. 0 | n = 0 |
| Jan 29, 2019 | Proquest D & T | 1. Dementia OR Alzheimer 2. Palliative OR end of life OR hospice 3. Rural 4. 1) & 2) & 3) | 1) 12099  2) 25464  3) 59532  4) 0 | n = 0 |
| **TOTAL** | | | **n = 12** | **n = 1** |
| **Targeted Website Search** | | | | |
| **Date** | **Organization name** | **URL**  **Search terms used within website** | **Items retrieved and screened for inclusion** | **Items included for synthesis** |
| Feb 6, 2019 | World Health Organization | <https://www.who.int/>  dementia, palliative/hospice/end of life | n = 15 | n = 1 |
| Feb 6, 2019 | Brain Exchange | <http://brainxchange.ca/>  rural | n = 12 | n = 0 |
| Feb 6, 2019 | Dementia Australia | <https://www.dementia.org.au/>  rural, palliative/hospice/end of life | n = 6 | n = 1 |
| Feb 6, 2019 | Dementia Alliance International | <https://www.dementiaallianceinternational.org/>  rural | n = 11 | n = 0 |
| Feb 6, 2019 | Dementia Care International | <https://dementiacareinternational.com/>  rural | n = 3 | n = 0 |
| Feb 6, 2019 | Alzheimer Society of Canada | <https://alzheimer.ca/en/>  rural | n = 8 | n = 0 |
| Feb 6, 2019 | Alzheimer’s Disease International | <https://www.alz.co.uk/>  rural, palliative/hospice/end of life | n = 9 | n = 0 |
| Feb 6, 2019 | Rural Health Information Hub | <https://www.ruralhealthinfo.org/>  dementia, palliative/hospice/end of life | n = 3 | n = 0 |
| **TOTAL** | | | **n = 67** | **n = 2** |
| **Google Web Search** | | | | |
| **Date** | **Search engine** | **Search strategy(s)** | **Items retrieved and screened for inclusion** | **Items included for synthesis** |
| Feb 6, 2019 | Google.ca (Advanced Search) | (intitle:palliative OR hospice OR end of life) (intitle:dementia) (intitle:rural) | **n = 68** | **n = 1** |
| **†Scholarly Database Search** | | | | |
| **Date** | **Databases** | **Search strategy(s)** | **Items retrieved and screened for inclusion** | **Items included for synthesis** |
| Nov 13, 2018 | MEDLINE, EMBASE, CINAHL, PSYCINFO | *See Appendix A for full electronic search strategies* | **n = 61** | **n = 8** |
| †Grey literature identified as potentially relevant during peer-reviewed literature search | | | | |
